# Secondary metabolites of *Alternaria*: A comprehensive review of chemical diversity and pharmacological properties

**DOI:** 10.3389/fmicb.2022.1085666

**Published:** 2023-01-06

**Authors:** Shiqin Zhao, Juan Li, Jinping Liu, Shaoyujia Xiao, Sumei Yang, Jiahui Mei, Mengyao Ren, Shuzhe Wu, Hongyuan Zhang, Xiliang Yang

**Affiliations:** ^1^Hubei Province Key Laboratory of Occupational Hazard Identification and Control, Department of Pharmacy, Institute of Infection, Immunology and Tumor Microenvironments, Institute of Pharmaceutical Process, Medical College, Wuhan University of Science and Technology, Wuhan, China; ^2^Department of Pharmacy, Tongji Hospital Affiliated to Tongji Medical College, Huazhong University of Science and Technology, Wuhan, China

**Keywords:** fungi, *Alternaria*, metabolites, bioactivity, biosynthesis, application

## Abstract

Fungi are considered to be one of the wealthiest sources of bio-metabolites that can be employed for yielding novel biomedical agents. *Alternaria*, including parasitic, saprophytic, and endophytic species, is a kind of dark fungi that can produce a broad array of secondary metabolites (SMs) widely distributed in many ecosystems. These are categorized into polyketides, nitrogen-containing compounds, quinones, terpenes, and others based on the unique structural features of the metabolites. New natural products derived from *Alternaria* exhibit excellent bioactivities characterized by antibacterial, antitumor, antioxidative, phytotoxic, and enzyme inhibitory properties. Thus, the bio-metabolites of *Alternaria* species are significantly meaningful for pharmaceutical, industrial, biotechnological, and medicinal applications. To update the catalog of secondary metabolites synthesized by *Alternaria* fungi, 216 newly described metabolites isolated from *Alternaria* fungi were summarized with their diverse chemical structures, pharmacological activity, and possible biosynthetic pathway. In addition, possible insights, avenues, and challenges for future research and development of *Alternaria* are discussed.

## 1. Introduction

Fungi are vital microorganisms that reside in various environments where they play a significant role in protecting eco-balance and diversity (Keller, 2019; Noor et al., 2020; Ibrahim et al., 2021). Fungi have attracted considerable attention in the fields of natural product chemistry, medicine, and agriculture (Al-Obaidi et al., 2021; Ibrahim et al., 2022). *Alternaria* fungus is a widespread dark fungus, belonging to classes Ascomycota, Dothideomycetes, Pleosporales, and Pleosporaceae (Feng and Sun, 2020). The fungal genus *Alternaria* is a ubiquitous group growing in diverse ecosystems worldwide as a parasitic, saprophytic, or endophytic species (Wang et al., 2022). Of these, *Alternaria alternata, Alternaria brassicicola, Alternaria penicillata, Alternaria cetera, Alternaria alternantherae*, and another 28 groups are ubiquitous (Feng and Sun, 2020; Li et al., 2021; Wang et al., 2022). *Alternaria* species can produce a variety of secondary metabolites. These metabolites mainly include polyketides, nitrogen-containing compounds, quinones, terpenes, and other compounds (Yamada et al., 2019; Li et al., 2020a; Tian et al., 2021). A large number of potentially bioactive molecules have been found, with intriguing structural skeletons and remarkable activities (Lou et al., 2013; Wang et al., 2022). Bioactive metabolites secreted by *Alternaria* fungi often exhibit excellent pharmacological potential, such as anticancer, antibacterial, antioxidant, and enzyme inhibitory effects (Wang J. et al., 2015; Dalinova et al., 2020; Mahmoud et al., 2021; Tian et al., 2021). For example, the world's first plant immune protein biological insecticide, ATailing, has been successfully developed by enhancing the broad-spectrum resistance of plants (Sheng et al., 2017). In addition, bio-metabolites of *Alternaria* fungi also have the efficacy of weeding and insecticide, and enhance the role of plant immunity in agricultural and food applications (Shi et al., 2017, 2018b; Tan et al., 2019; Li et al., 2021).

**Figure 1:**
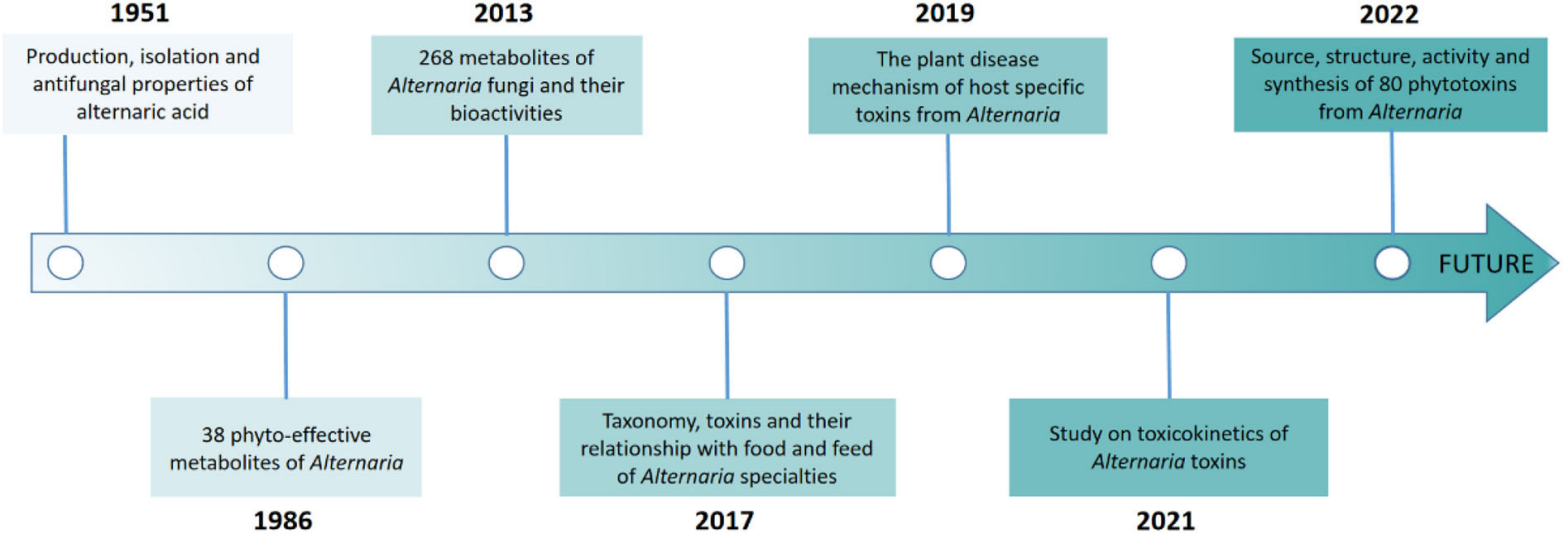
Timeline of related studies on the bio-metabolites of *Alternaria* fungi.

Furthermore, continuous studies on *Alternaria* metabolites have been carried out on the production, isolation, chemical complexity, culture conditions, plant disease mechanisms and toxicokinetics of toxin metabolomics (Figure 1) (Brian et al., 1951; Bemmann, 1986; Pinto and Patriarca, 2017; Sheng et al., 2017; Meena and Samal, 2019; Chen et al., 2021). A recent review focused on the 80 *Alternaria* phytotoxins with their classification, chemical structure, occurrence, bioactivity, and biosynthesis (Wang et al., 2022). These metabolites have an important but less-explored application value in the microorganism, where the chemical industry and fields of medicine, biological control, etc. have endeavored to discover structurally novel natural products. In this study, we summarize the new *Alternaria*-derived metabolites and give a general overview of the occurrence, chemical structure, and pharmacological properties of secondary metabolites as seen in research from 2014 to 2022. In addition, biosynthetic pathways with some biologically important metabolites are also discussed, which provide new research opportunities for the discovery of drug compounds and practical production technology in the future. Related literature can be found on various databases, including Science Direct, PubMed, Elsevier, Google Scholar, Baidu Scholar, CNKI, and Springer.

## 2. Secondary metabolites of *Alternaria* fungi

### 2.1. Polyketides

**Figure 2:**
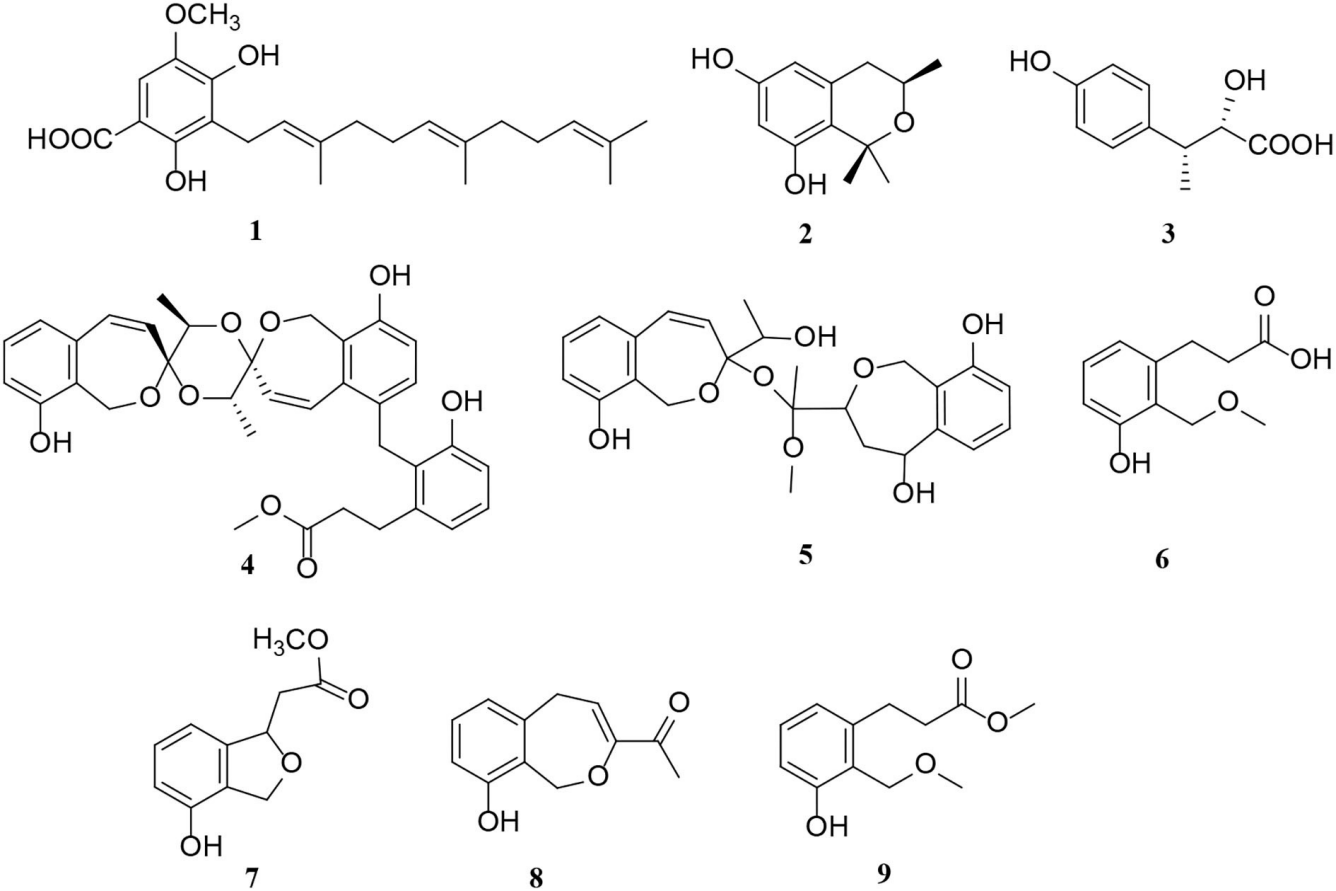
Simple phenylpropanoid derivatives (**1**–**9**) from *Alternaria* fungi.

**Figure 3:**
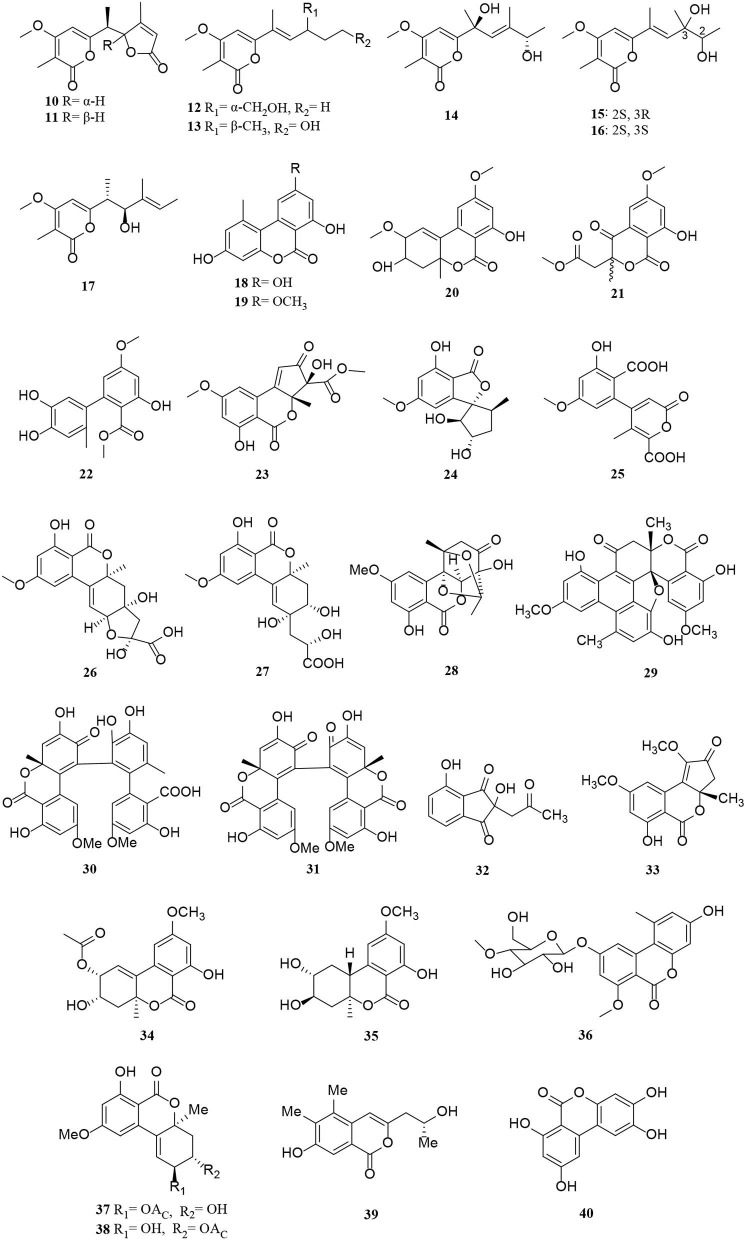
Pyranone derivatives (**10**–**40**) from *Alternaria* fungi.

**Figure 4:**
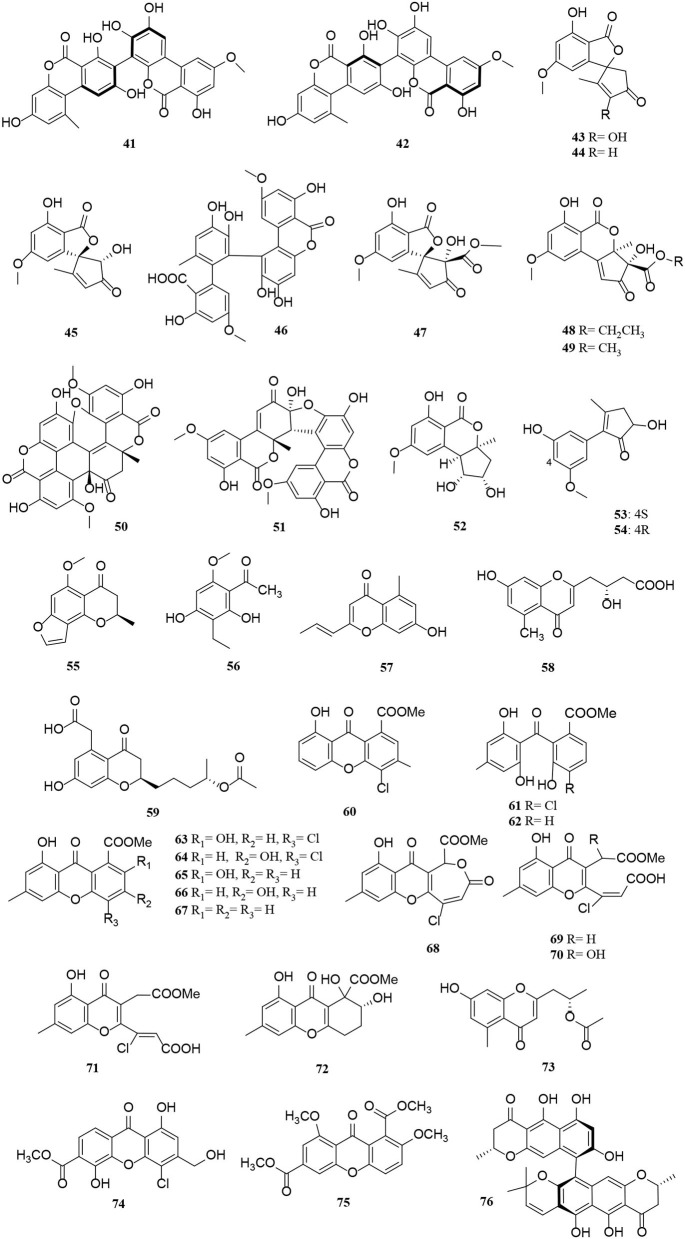
Pyranone derivatives (**41**–**76**) from *Alternaria* fungi.

**Figure 5:**
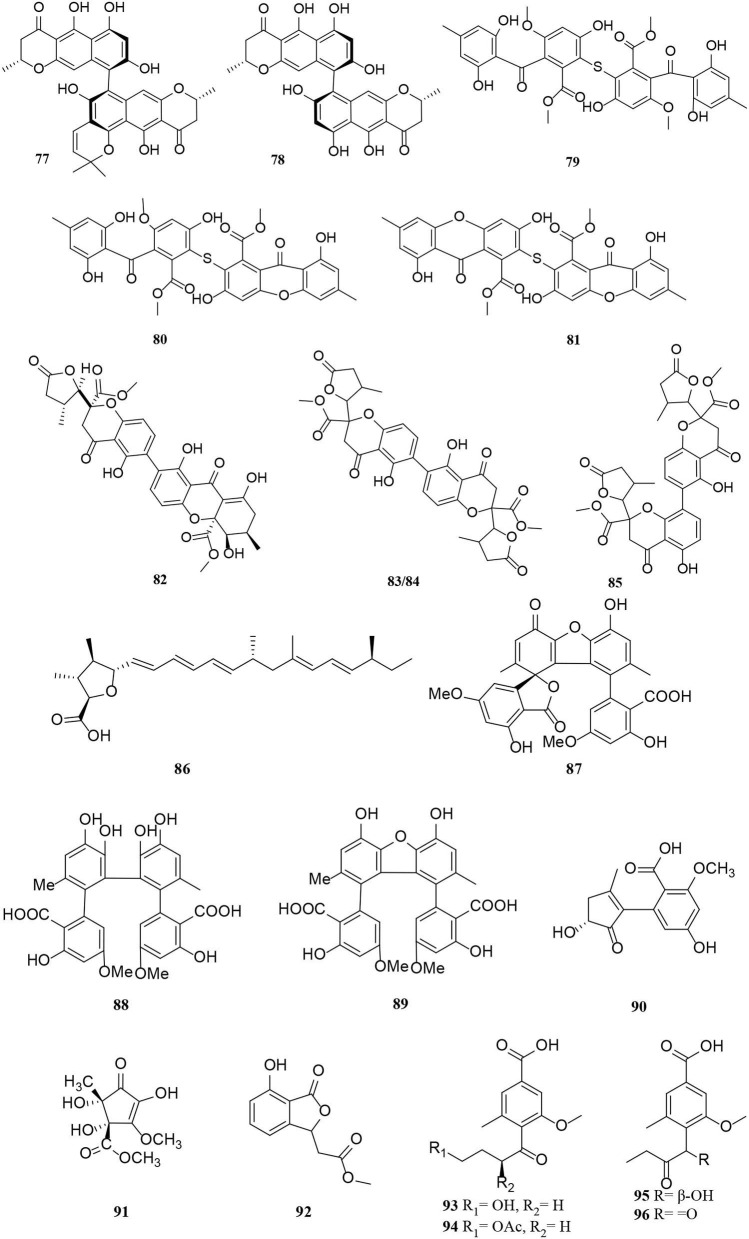
Polyketide derivatives (**77**–**96**) from *Alternaria* fungi.

Polyketides are potential virulence factors and immunosuppressants. Pathogenic fungi, which can be synthesized from simple acyl building blocks, exhibit a high degree of structural diversity (Miyanaga, 2017). Polyketides are important natural metabolites that have attracted considerable attention. Simple phenylpropanoids and pyranones are the major groups of the polyketide family secreted by *Alternaria* sp. A total of 96 polyketides, 9 simple phenylpropanoids (**1**–**9**) (Figure 2), 76 pyranones (**10**–**85**) (Figures 3, 4), and 11 other polyketides (**86**–**96**) (Figure 5) are summarized. Most pyranones have intriguing stereoisomeric frameworks, which are described in detail in this article.

Simple phenylpropanoids are also common in *Alternaria* endophytes (Figure 2). A total of nine novel phenylpropanoid derivatives, namely alternaritins B–C (**1**–**2**), (2S, 3R)-2-hydroxy-3-(4-hydroxyphenyl) butanoic acid (**3**), and alternarias A–F (**4**–**9**), were isolated from the *Alternaria* species (Lu et al., 2021; Tian et al., 2021). Notably, alternaritin C (**2**), composed of hydrogenated pyran and tetrasubstituted benzene, is a rare carbon skeleton with double-ring units (Tian et al., 2021). In addition, compound **3** was a new natural product consisting of a p-substituted phenol moiety and a 2-hydroxybutyric acid fragment (Tian et al., 2021).

Pyranones, also known as pyrones, include α-, β-, and γ-pyranones. Most pyranones isolated from *Alternaria* fungi belong to α-pyranones, and most of these have enantiomeric structures, including dibenzo-α-pyranone derivatives (Tang et al., 2019), aromatic polyketone dimers (Yang C. L. et al., 2019), cyclopentane isochromone derivatives (Lu et al., 2018), and biphenyl structure derivatives (Kong et al., 2020). Of these, three pairs of unprecedented α-enantiomers of pyrone derivatives (**10**–**12**) were derived from *Alternaria brassicicola*, along with five diastereomeric structures, alterpyrones D-H (**13**–**17**) (Li et al., 2021). Structurally, two pyranone derivatives, alternariol (**18**) and alternariol-9-methyl ether (**19**), isolated from the marine endophytic *Alternaria*, have the same tricyclic skeleton as the alternates A–C (**20**–**22**) (Mahmoud et al., 2021; Wang et al., 2021). In addition, alternatiol (**23**) was reported as a new altenusin metabolite separated from *Vitex rotundifolia Alternaria alternata* JS0515 (Lee et al., 2019). Alternatains A–D (**24**–**27**) were obtained from the solid substrate cultures of *Alternaria alternata* MT-47 (Yang H. et al., 2019). It can be inferred that it is mainly composed of acetyl coenzyme A and polyketone synthase according to structural characteristics (Yang H. et al., 2019). The enantiomer (+)- and (–)- alternarilactone A (**28**) was identified as a dibenzo-α-pyranone derivative, possessing a diepoxy-cage-like moiety isolated from *Alternaria* sp. Hh930. (Tang et al., 2019). Interestingly, (+)- and (–)-alternamgin (**29**) is also an enantiomeric pyranone derivative with an unprecedented 6/6/6/6/5/6/6 seven-ring framework from *Vitis quinquangularis* (Wu J. C. et al., 2019). A new example of aromatic polyketone dimer metabolite, bialternacins E-F (**30**–**31**), was produced by *Alternaria* sp. NF2128 from the stem of *Maianthemum bifolium* fungus (Yang C. L. et al., 2019). Notably, indandione B (**32**), featuring an extremely rare indole ketone moiety, was found in the *Morinda officinalis* fungus *Alternaria* sp. A744 (Wang et al., 2017). The absolute configuration of compound **33** was determined as a pair of new cyclopentane isochromone enantiomers by 2D-nuclear magnetic resonance (2D-NMR) and high-resolution electrospray ionization mass spectroscopy (HRESIMS) (Lu et al., 2018). Compounds **34**–**42** possess a similar three-ring system, formed an ester bond between a six-membered ring and phenol. Interestingly, the third ring of **39** is open, and both **41** and **42** are dimers (Wang et al., 2014; Xu et al., 2015; Tian et al., 2017; Kong et al., 2020). The *Alternaria alternata* ZHJG5 produced a series of compounds (**43**–**49)**, including five novel polyketide derivatives (**43**–**46**) and three pairs of dibenzo-α-pyrone derivatives (**47**–**49**) (Zhao et al., 2020, 2021). In this study, (±) alternarlactones A (**50**) and B (**51**) were two new isolated dimers, which were formed by the C-O- and C-C-bond between dehydroaltenusin and alternariol from *Halophyte Salicornia* sp. fungus *Alternaria alternata* P1210 (Shi et al., 2019). In addition, the isolation of the same marine fungi *Alternaria* sp. SCSIO41014 yielded three new α-pyranone derivatives (**52**–**54**) (Pang et al., 2018). Compounds **53** and **54** were proved to be two stereoisomeric configurations isolated from marine sponge (Pang et al., 2018).

Two new phomalone derivatives, phomalichenones E-F (**55**–**56**), were isolated from a deep-sea-derived fungus, *Alternaria* sp. MCCC 3A00467 (Zhong et al., 2022). **56** is an open γ-pyranone ring with an acetyl group at C-1 compared with **55**. Alterchromanone A (**59**) is a new chromanone derivative, also isolated from marine *Alternaria longipes* (Liu et al., 2021). Structurally, alternate D (**57**) and alternaritin D (**58**) have similar benzo-γ-pyranone moiety (Tian et al., 2021; Wang et al., 2021). A total of 13 compounds (**60**–**72**) were isolated from *Alternaria sonchi*, including chromones, xanthones, and benzophenones (Dalinova et al., 2020). Among them, **60** and **61** represent two new derivatives of chlorinated anthrone and benzophenone, respectively, which were determined by spectroscopy (mainly through 2D-NMR and MS). And compounds **62**, **64**–**67**, **71**, and **72** were first reported for *Alternaria sonchi* (Dalinova et al., 2020). In addition, (2′S)-2-(2-acetoxypropyl)-7-hydroxy-5-methylchromone (**73**) was isolated from the *Vitex rotundifolia* endophytic fungus *Alternaria brassicae* JS959 (Kim et al., 2019). Compounds (**74**–**75**) with xanthone moiety were isolated from the marine *Alternaria* sp. R6 (Wang J. et al., 2015). 4-chloro-1,5-dihydroxy-3-hydroxymethyl-6-methoxycarbonyl-xanthen-9-one (**74**) bearing a chlorine atom was also named 4-chlorofischexanthone. Two new cephalochromin derivatives, prenylcephalochromin A (**76**) and prenylcephalochromin B (**77**), along with cephalochromin (**78**), were isolated from the *Dasymaschalon rostratum* fungus *Alternaria* sp. ZG22 (Song et al., 2021). Notably, **76** were elucidated by comprehensive spectroscopic methods, indicating that **76** bears a bis-naphtho-γ-pyrone skeleton. Polluxochrin (**79**) and dioscin (**80**), two new dimers of sulochrin linked by thioether bonds, as well as another five compounds (**81**–**85**), were purified from an *Alternaria* sp. isolate obtained from Hawaiian soil (Cai et al., 2014). Compounds **80**–**81** were produced by intramolecular cyclization of **82**, and metabolites **82**–**85** were four secalonic acid analogs (Cai et al., 2014). Compound **83** is a symmetrical dimer. Overall, the planar structure of **83**, especially the C-6–C-6′ linkage, was established by the HMBC correlation spectrum. Subsequently, **84** was determined to share the same planar structure as **83**. However, the presence of two distinct sets of resonances representing the two monomeric portions of **84** denoted it was an asymmetric diastereomer of **83**.

Other polyketides include aliphatic polyketone (**86**), aromatic polyketone dimer (**87**–**89**), and alternative acid B (**90**) (Ding et al., 2017; Xu et al., 2019; Yang C. L. et al., 2019). One new cyclohexanone derivative with unsaturated ketone groups was (±)-(4S^*^,5S^*^)-2,4,5-trihydroxy-3-methoxy-4-methoxycarbonyl-5-methyl-2-cyclopentene-1-one (**91**), which was characterized to originate from the mangrove *Alternaria* strain (Wang J. et al., 2015). Isobenzofuranone A (**92**) bearing isobenzofuranone moiety was isolated from the *Morinda officinalis* fungus *Alternaria* sp. A744 (Wang et al., 2017). Finally, four new pyrenochaetic acid derivatives (**93**–**96**) isolated from soil samples have the same carbon skeleton by analysis of the ^1^H and ^13^C NMR data (Cai et al., 2014).

### 2.2. Nitrogen-containing compounds

**Figure 6:**
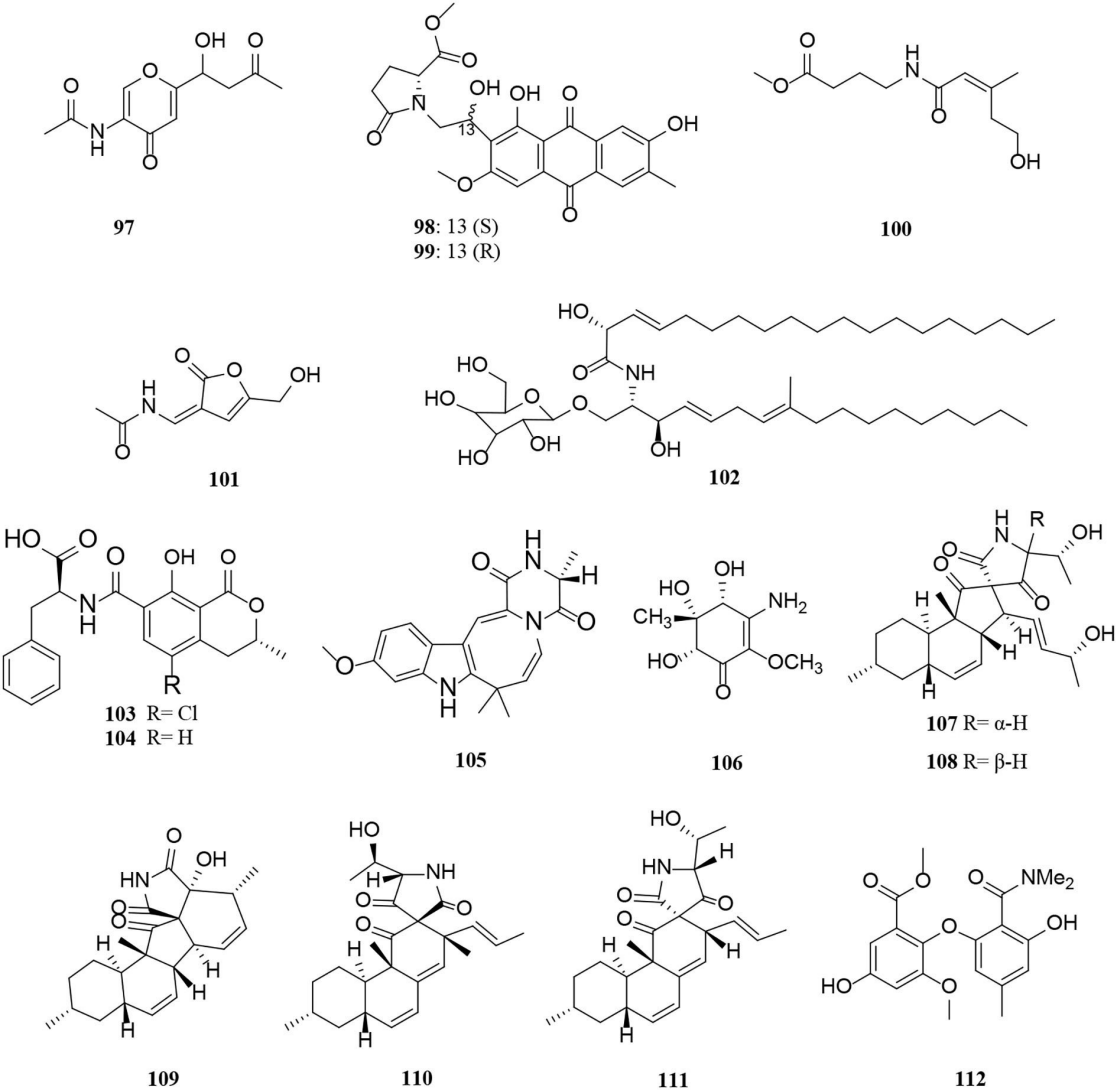
Amide derivatives (**97**–**112**) from *Alternaria* fungi.

**Figure 7:**
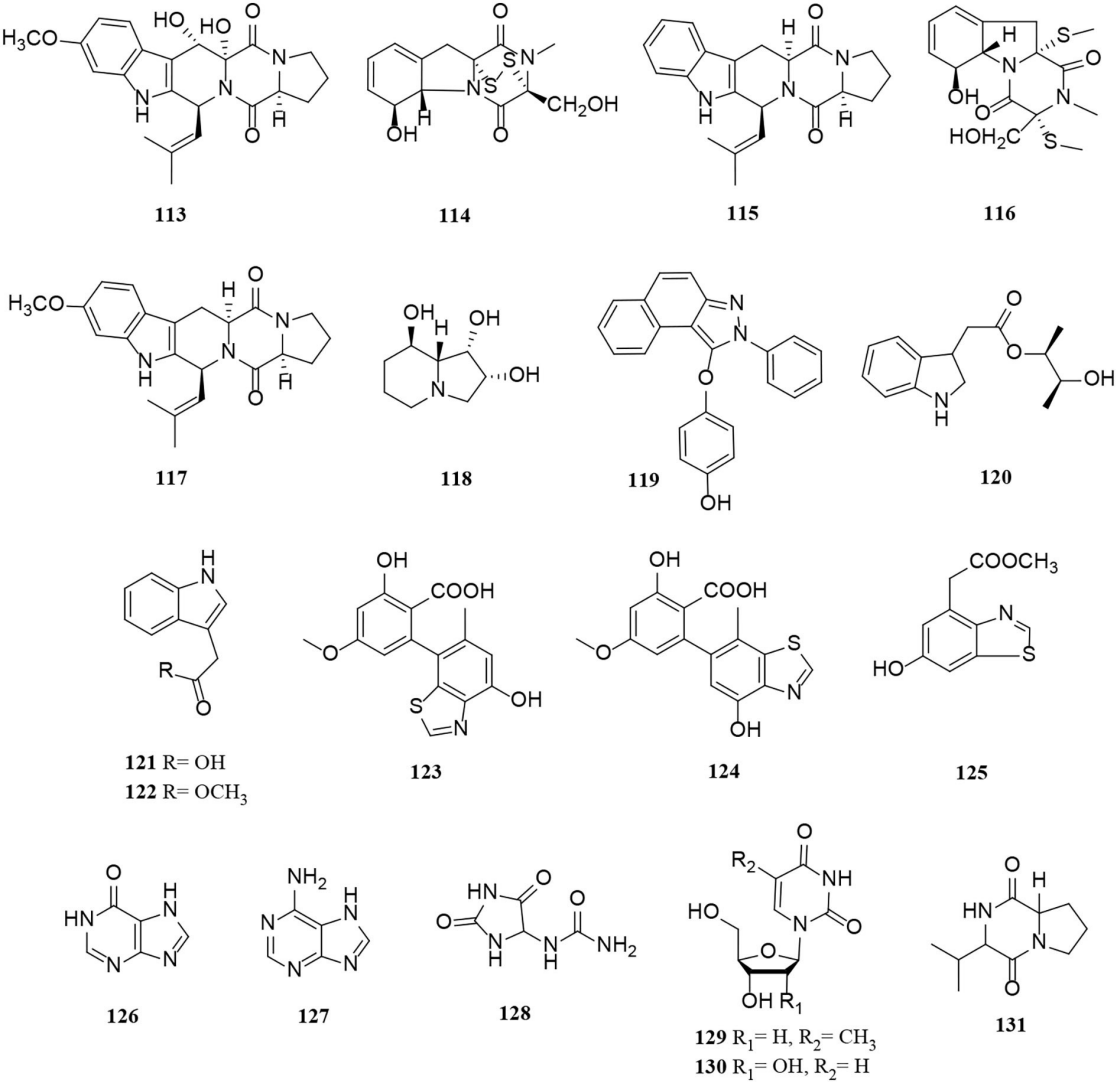
Peptide and alkaloid derivatives (**113**–**131**) from *Alternaria* fungi.

**Figure 8:**
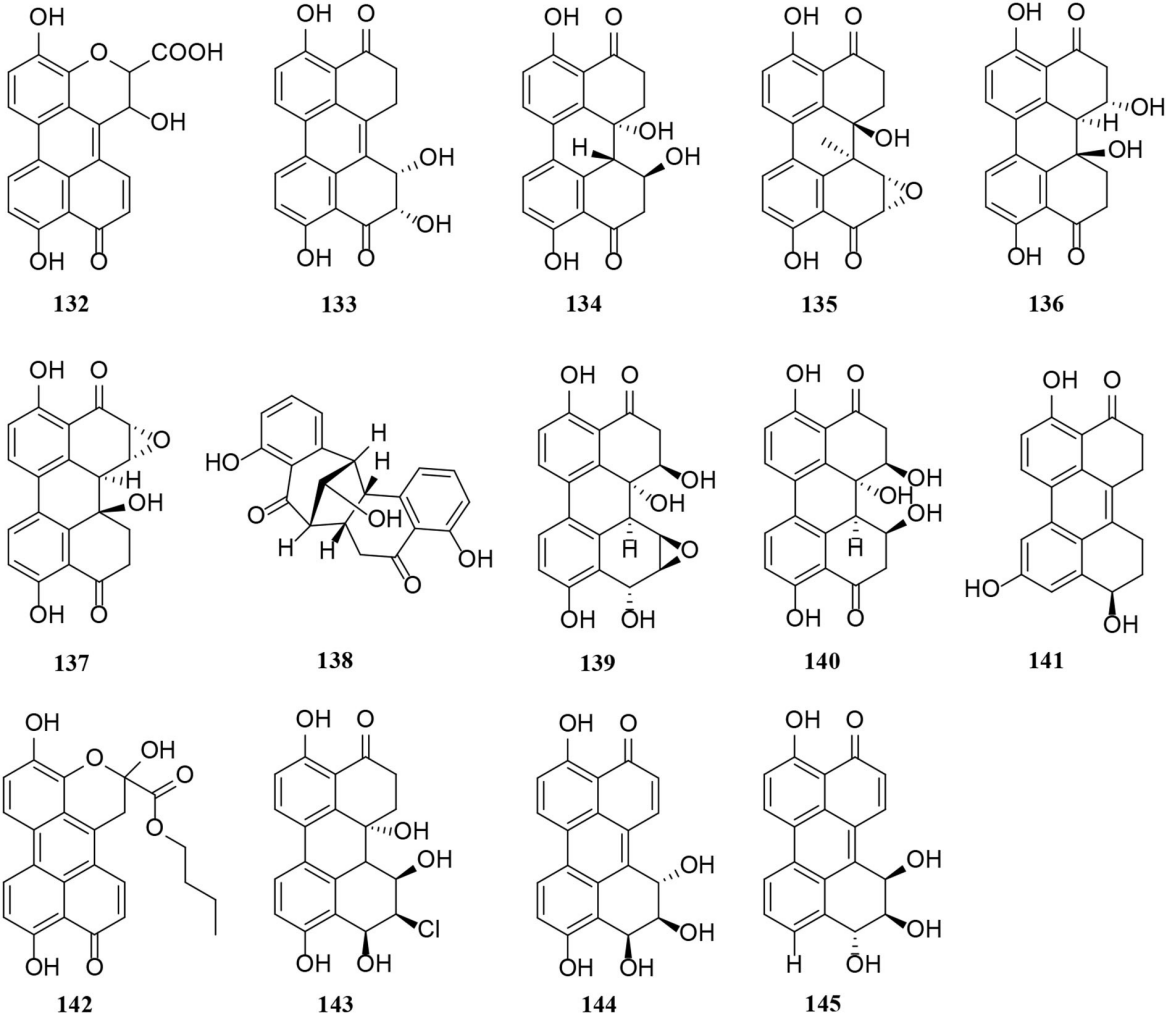
Perylene quinone derivatives (**132**–**145**) from *Alternaria* fungi.

Nitrogen-containing compounds, isolated from *Alternaria*, include amides, peptides, and alkaloids. A total of 35 nitrogen-containing compounds, 16 amides (**97**–**112**) (Figure 6), 5 peptides (**113**–**117**) (Figure 7), and 14 alkaloids (**118**–**131**) (Figure 8) have been summarized and are described in detail as follows.

A pair of enantiomeric nitrogen-containing compounds, alternaritin A [(±)-**97**], is composed of the amide bond and γ-pyranone composition isolated from *Alternaria* sp. MG1 (Tian et al., 2021). Structurally, two new anthraquinones named anthrininones B-C (**98–99**) with a 4,5-disubstituted butylaminolate unit were obtained from the marine fungus *Alternaria tenuissima* DFFSCS013 (Pan et al., 2019). In addition, alteamide (**100**) bearing oxygenated prenyl group was obtained from *Alternaria alternata* (Wang et al., 2021). 2-(N-vinylacetamide)-4-hydroxymethyl-3-ene-butyrolactone (**101**) and chrysogeside F (**102**) were isolated from a marine-derived fungus *Alternaria* sp. NH-F6 bearing 3-ene-butyrolactone moiety and methyl D-glucopyranoside moiety structures, respectively (Ding et al., 2017). Compounds **103**–**106** were amide derivatives extracted from marine microorganisms (Li et al., 2015; Wang J. et al., 2015). Of these, (±)-(4R^*^,5S^*^,6S^*^)-3-amino-4,5,6-trihydroxy-2-methoxy-5-methyl-2-cyclohexen-1-one (**106**) is a new cyclohexenone derivative isolated from the marine *Nerium indicum Alternaria* sp. SPS-04 (Wang J. et al., 2015). Five new decalin derivatives, altercrasins A-E (**107–111**), contain lactam-ring structures from a sea-urchin-derived *Alternaria* sp. (Yamada et al., 2019). The absolute stereostructure of altercrasins A (**107**) was determined by NMR chemical shifts, NOESY correlations, and electronic circular dichroism (ECD) spectral analyses, and furthermore deduced by chemical transformation and the modified Mosher's method. As a result, the compound pairs of **107/108** and **110/111** were ascertained to be stereoisomers, deduced by the aforementioned methods respectively (Yamada et al., 2019). Dimethylamide asterrate (**112**), one new asterric acid analog with two new methyl groups, was obtained from an *Alternaria* sp. isolate (Cai et al., 2014).

Diketopiperazines (DKPs) consisting of two α-amino acids and cyclic dipeptides are amino acid peptides (He et al., 2019). Five new diketopiperazine derivatives (**113**–**117**) were isolated from the marine *Alternaria alternate* HK-25 (He et al., 2019). In comparison with conventional column chromatography with either C18 or C8 columns, compounds **114** and **116** were successfully separated from crude samples by a new high-speed counter-current chromatography (HSCCC) elution method with high efficiency and recovery (He et al., 2019).

Most alkaloids have heterocyclic structures, such as swainsonine (**118**), 2H-benzindazole derivative (**119**), indole derivatives (**120**–**122**), and thiazoles (**123**–**125**) (Chen et al., 2018; Tan et al., 2019; Wu J. C. et al., 2019; Xu et al., 2019). Alterindazolin A (**119**) is a rare heterocyclic aromatic compound, containing indazole from *Alternaria alternata* Shm-1 (Wu X. et al., 2019). Similarly, altenusinoide A (**123**) and altenusinoide B (**124**) have an unusual altenusin-thiazole-fused skeleton core (6/6/5) (Chen et al., 2018). Moreover, compound **125** was identified as the first benzothiazole secondary metabolite from the marine sponge-derived fungus *Alternaria* sp. SCSIOS02F49 (Chen et al., 2018). Compounds (**126**–**130**) were purine and pyrimidine derivatives from different *Alternaria* strains (Miao et al., 2017). Compound (**131**) was a maculosin derivative isolated from *Alternaria alternata* (Hawas et al., 2015).

### 2.3. Quinones

**Figure 9:**
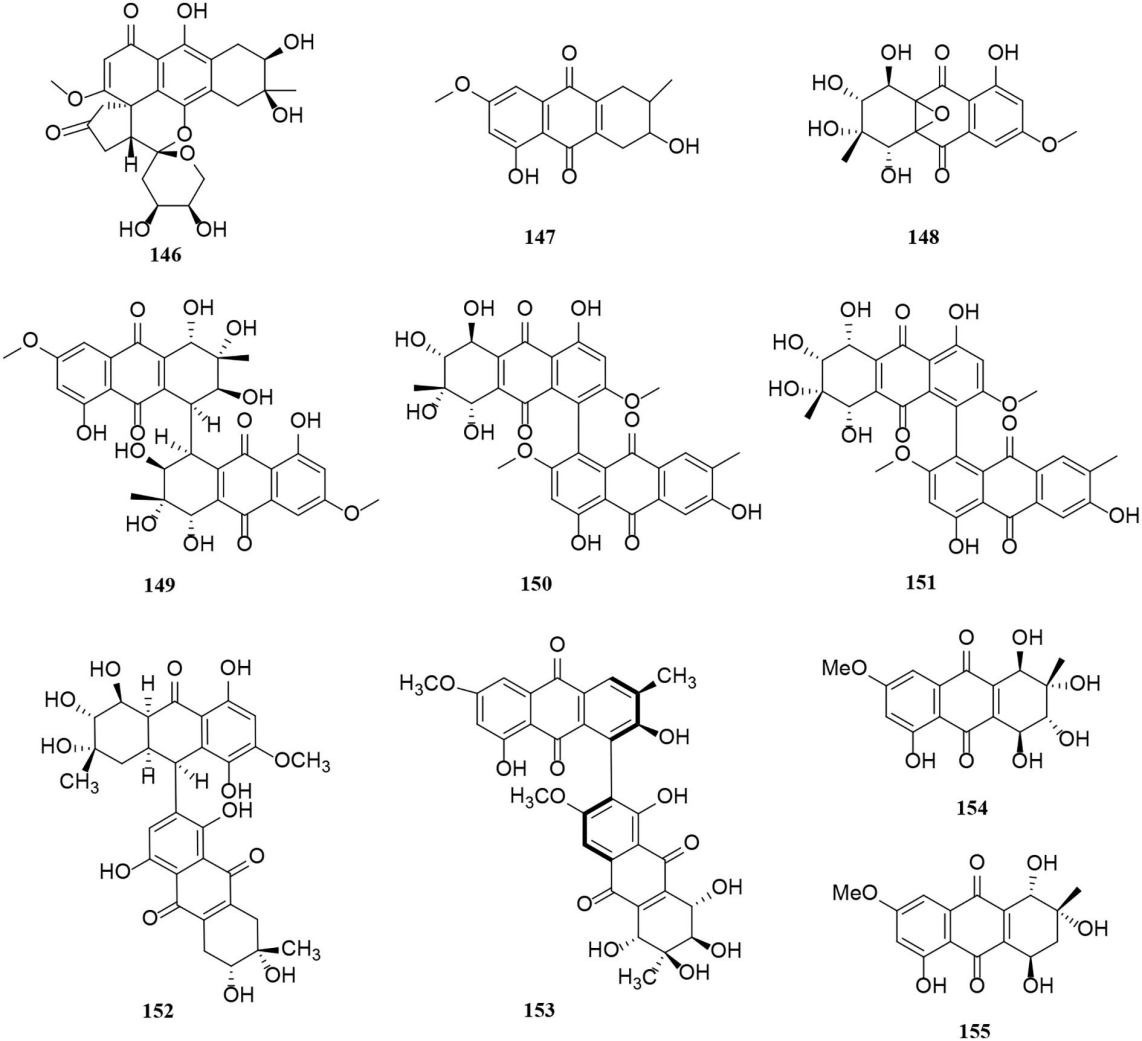
Anthraquinone derivatives (**146**–**155**) from *Alternaria* fungi.

**Figure 10:**
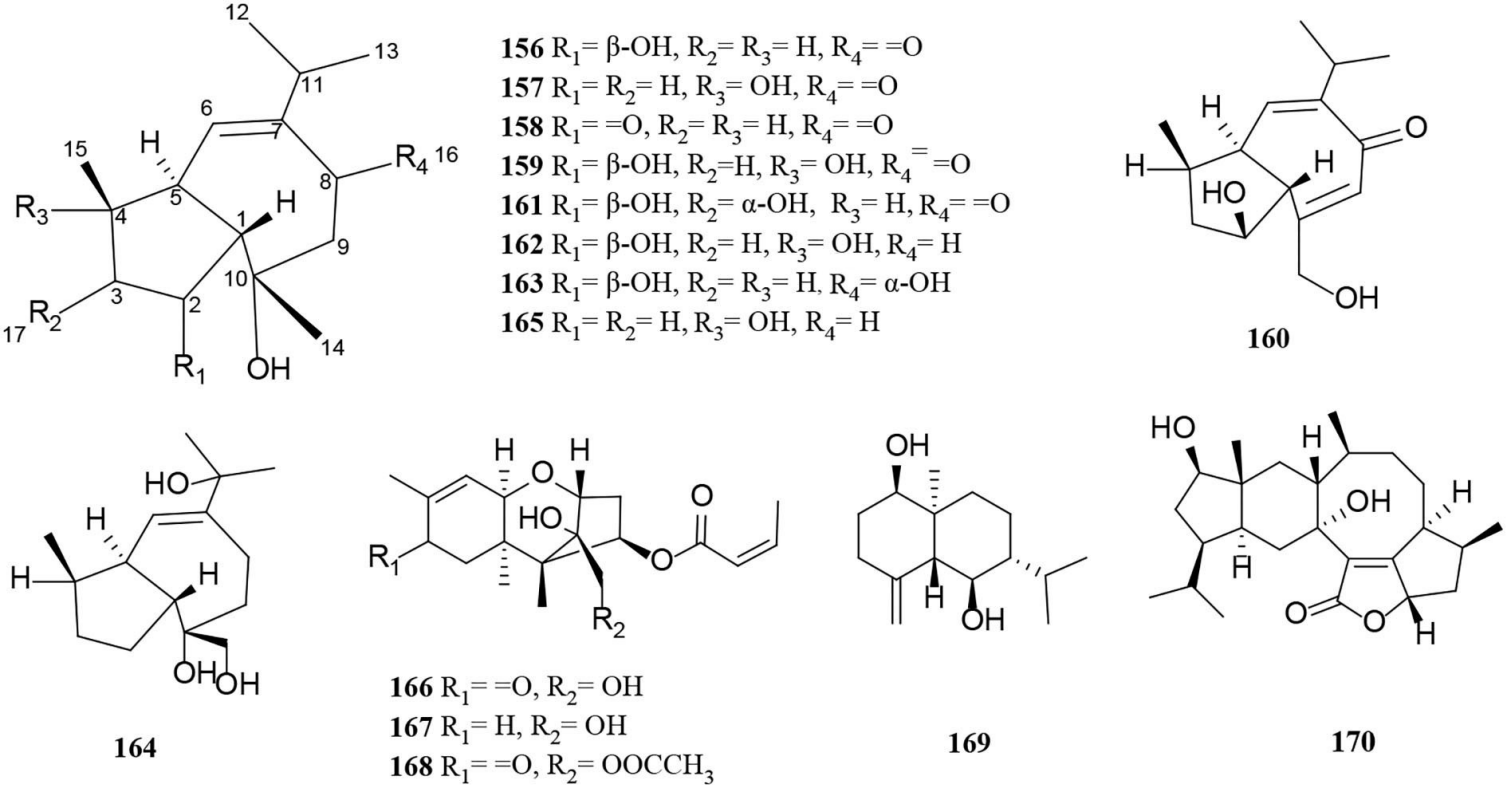
Sesquiterpene derivatives (**156**–**170**) from *Alternaria* fungi.

So far, there are two groups of quinones among *Alternaria* metabolites that have been isolated, perylenequinones and anthraquinones. In this part of the research, 14 perylenequinones (**132**–**145**) (Figure 8) and 10 anthraquinones (**146**–**155**) (Figure 9) were produced. Perylenequinones are a class of highly conjugated pentacyclic nuclear aromatic polyketones, which are described in detail as follows (Zhao et al., 2019).

Perylenequinone is generally a dark-colored pigment characterized by an oxidized pentacyclic nuclear skeleton and has been widely used in traditional Chinese herbal medicine (Tantry et al., 2018). Four compounds (**132**–**135**) also have the structural skeleton of perylene quinone, namely isoxanalteric acid I (**132**), altertoxin VII (**133**), altertoxin I (**134**), and altertoxin II (**135**) (Kong et al., 2020; Mahmoud et al., 2021; Tian et al., 2021). In addition, altertoxin I (**136**) and altertoxin II (**137**) are two perylene quinone cytotoxins from *Alternaria alternata* (Hohenbichler et al., 2020). A novel perylenequinone-related derivative, known as alternatone A (**138**), was isolated from the marine *Alternaria alternata* L3111′, which possessed an unprecedented tricyclo [6.3.1.0] dodecane skeleton (Zhao et al., 2019). Furthermore, two new perylenequinones (**139**–**140**) were isolated from the *Pinus ponderosa* endophytic *Alternaria* sp. (Tantry et al., 2018). Compared with compound **140**, compound **139** has a significantly epoxide ring. Notably, altertoxin VII (**141**) and butyl xanalterate (**142**) are two new polyketides from the sponge-derived fungus *Alternaria* sp. SCSIO41014. And **141** is the first example to bear a novel 4,8-dihydroxy-substituted perylenequinone structure, while the phenolic hydroxy groups be commonly substituted at C-4 and C-8 (Pang et al., 2018). Moreover, two new perylenequinones (**143**–**144**) have a similar structure to deep-sea sediment fungus *Alternaria* sp. NH-F6, which is characterized as a tetrahydroperylenone (Ding et al., 2017). Altertoxin IV (**145**) is also a new tetrahydroperylene ketone derivative from the *Broussonetia papyrifera* fungus *Alternaria* species G7 (Zhang et al., 2016).

A novel hydroanthraquinone, anthrininone A (**146**), possessing an unprecedented hexacyclic spiro-fused ring skeleton, was isolated from the marine fungus *Alternaria tenuissima* DFFSCS013 (Pan et al., 2019). In addition, macrosporin (**147**) is an anthraquinone from marine *Alternaria* species (Wang Y. N. et al., 2015). Four new anthraquinone derivatives, compounds (**148**–**151**), were isolated from the saline lake *Alternaria* sp. XZSBG-1 (Chen et al., 2014). In this study, altersolanol O (**148**) and alterporriol S (**149**) are relatively rare compounds, representing a novel tetrahydroanthraquinone bearing an epoxy ether bond between C-4a and C-9a and a tetrahydroanthraquinone dimer bearing a C-4-C-4' linkage, respectively (Chen et al., 2014). Alterporriol S (**152**) and (+)-aS-alterporriol C (**153**) were also obtained from the marine *Alternaria* sp. SK11 (Xia et al., 2014). A novel alterporriol-type anthranoid dimer, alterporriol S (**152**), was represented as the first member of the alterporriol family to possess a unique C-10–C-2′ linkage (Xia et al., 2014). In addition, two anthraquinones (**154**–**155**) were isolated from the endophyte *Alternaria* sp. in *Erythrina variegata* (Pompeng et al., 2013).

### 2.4. Terpenes

**Figure 11:**
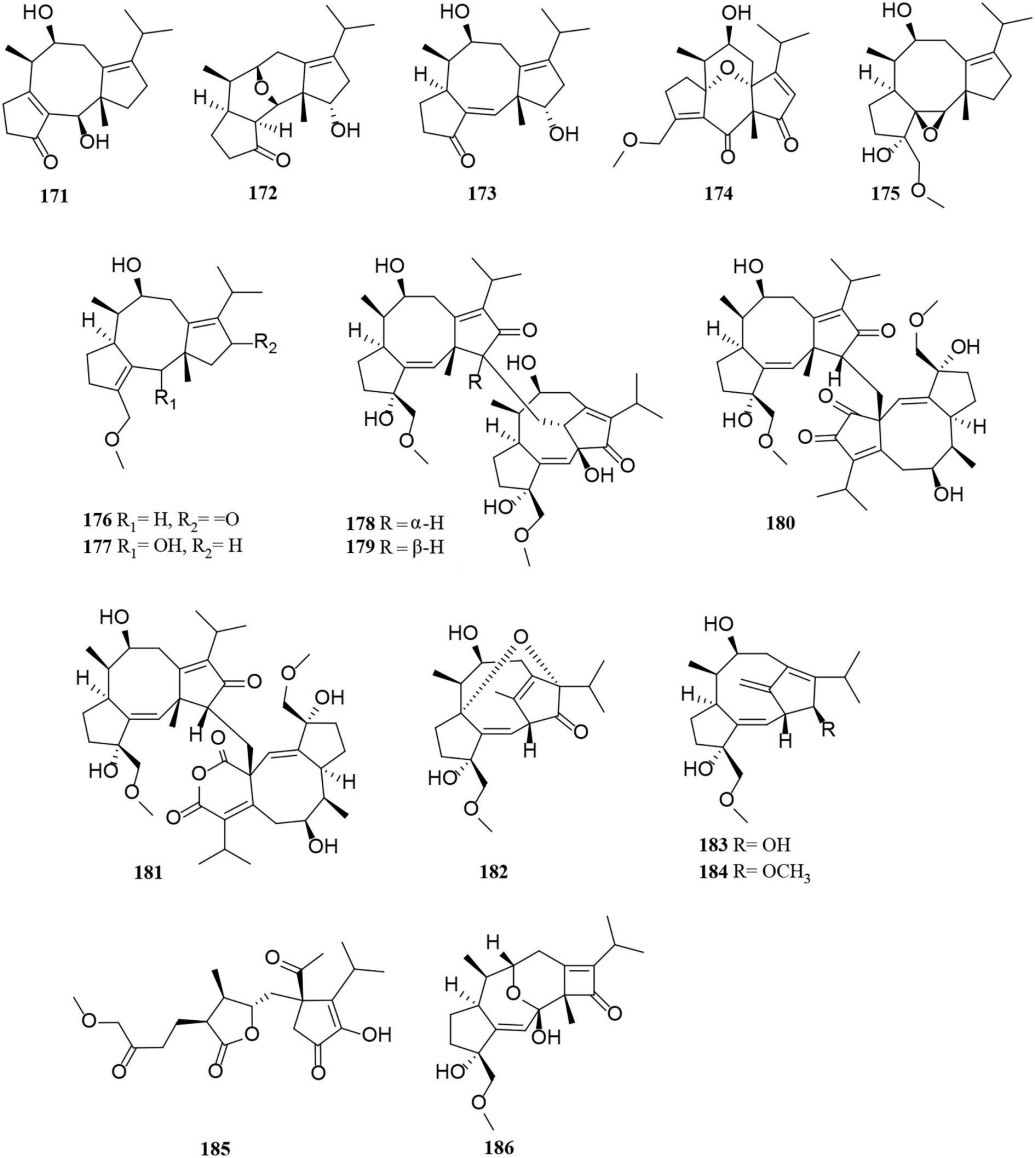
Diterpene derivatives (**171**–**186**) from *Alternaria* fungi.

**Figure 12:**
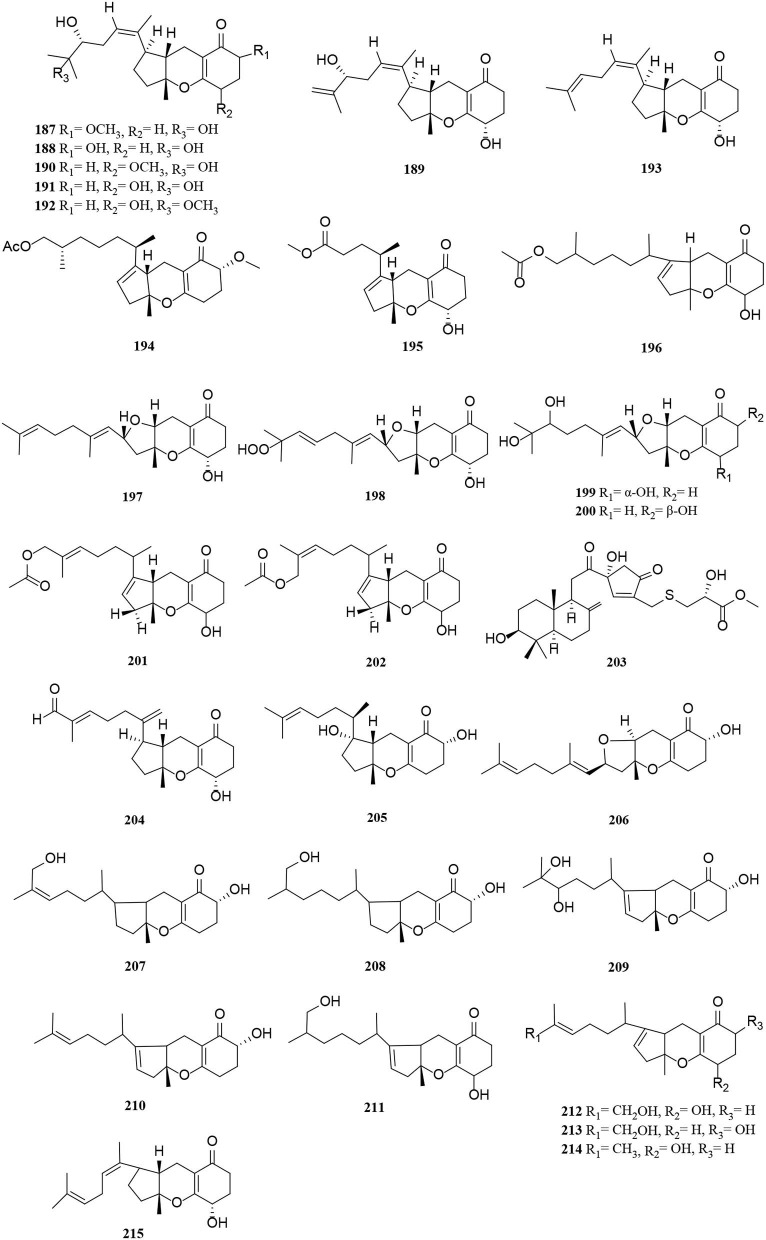
Meroterpenoid derivatives (**187**–**215**) from *Alternaria* fungi.

Terpenoids from *Alternaria* fungi include sesquiterpenes, diterpenes, and meroterpenoids. In this section, a total of 60 terpenoids, comprising 15 sesquiterpenes (**156**–**170**) (Figure 10), 16 diterpenes (**171**–**186**) (Figure 11), and 29 meroterpenoids (**187**–**215**) (Figure 12), are summarized. The specific description is as follows.

Oxytropiols A-J (**156**–**165**) were found in 10 undescribed guaiane-type sesquiterpenoids isolated from *Alternaria oxytropis* (Tan et al., 2019). Their typical structural feature is that they construct a seven-membered ring and fuse a five-membered ring, indicating a guaiacol-type sesquiterpene skeleton. New trichothecene derivatives with a 1, 2-diol moiety at C-12 and C-13, alterchothecenes A-C (**166**–**168**), were isolated from *Alternaria* sp. sb23 bearing a 12, 13-epoxytrichothec-9-ene ring moiety (Gao et al., 2020). Spectra data analysis of NMR, DEPT, and HSQC suggested that **167** is 8-dihydrogeneated derivatives and **168** is 13-acetylated derivatives of **166** respectively (Gao et al., 2020). Similarly, (1R,5R,6R,7R,10S)-1,6-Dihroxyeudesm-4(15)-ene (**169**) is a new sesquiterpenoid isolated from *Alternaria alternate* (Xu et al., 2019). In addition, sesteralterin (**170**) represents the first nitidasane sesterterpene obtained from the marine *Alternaria alternata* strain (k21-1) (Shi et al., 2017).

Compounds (**171**–**177**) were new fusicoccane-like diterpenoids isolated from modified rice cultures medium of *Alternaria brassicicola*, among which compounds (**171**–**173**) possess a rare 16-nor-dicyclopenta [a, d] cyclooctane structure, compounds **172** and **174** feature two previously new tetracyclic 5/6/6/5 ring systems that represent the typical examples of fusicoccane-type diterpenoids, and compound **175** features a new tetracyclic 5/8/5/3 ring system (Li et al., 2020a). Interestingly, four unprecedented diterpene dimers, alterbrassinoids A-D (**178–181**), were obtained in the same manner as above (Li et al., 2019a). Compounds (**178–181**) are the first examples of fusicoccane-derived diterpenoid dimers furnished by forming an undescribed C-12–C-18′ linkage, in which **178** and **179** represent unprecedented heterodimers, whereas **180** and **181** represent unprecedented homodimers (Li et al., 2019a). This suggests that the production of new compounds can be achieved by modifying the medium (Li et al., 2019a, 2020a). Three new rearranged fusicoccane diterpenoids, alterbrassicenes B–D (**182–184**) bearing a rare bridgehead double-bond-containing tricyclo [9.2.1.0] tetradecane core skeleton found from *Alternaria brassicicola* (Li et al., 2020b). A highly functionalized diterpenoid, alterbrassicicene A (**185**), with a new monocyclic carbon skeleton bearing unique dihydro-2(3H)-furanone and 2-cyclopenten1-one motifs, was obtained from *Alternaria brassicicola* (Li et al., 2018). Alterbrassicene A (**186**) was characterized as a fusicoccane-derived diterpenoid, possessing an undescribed 5/9/4-fused carbocyclic framework bearing a rare 2-cyclobuten-1-one motif, which were obtained from *Alternaria brassicicola* (Hu et al., 2018).

The new compounds (**187–196**) have a similar tricycloalternarene structure to each other (Shen et al., 2018; Shi et al., 2018a; Li et al., 2019b). Of these, tricycloalternarenes Q-W (**187–193**) were characterized as seven unprecedented metabolites from *Alternaria brassicicola* (Li et al., 2019b). Four new meroterpenes, tricycloalterfurenes A-D (**197–200**), rarely occur in tricycloalternarenes and bear a tetrahydrofuran unit obtained from an *Alternaria alternata* strain (k21-1). Compound **199** represents the first hydroperoxy-containing tricycloalternarene (Shi et al., 2017). Two new 15-hydroxytricycloalternarenes (**201–202**) represent a pair of E and Z isomers, possessing a double bond linked by an acetoxymethylene group (Shi et al., 2018a). A rearranged drimane meroterpenoid with a thioglycerate moiety, alternarin A (**203**), was obtained from the marine fungi *Alternaria* sp. ZH-15 (Wang H. L. et al., 2020). Tricycloalternarenes X-Y (**204–205**) and metabolites (**206–211**) were meroterpenoid compounds isolated similarly from the marine fungi (Pan et al., 2018; Wang L. et al., 2020). Compounds **212–215** were mixed terpenoids isolated from the endophyte *Alternaria* sp. Be-1 of the insect Pierisrapae Linne (Zhang et al., 2015).

### 2.5. Other classes

**Figure 13:**
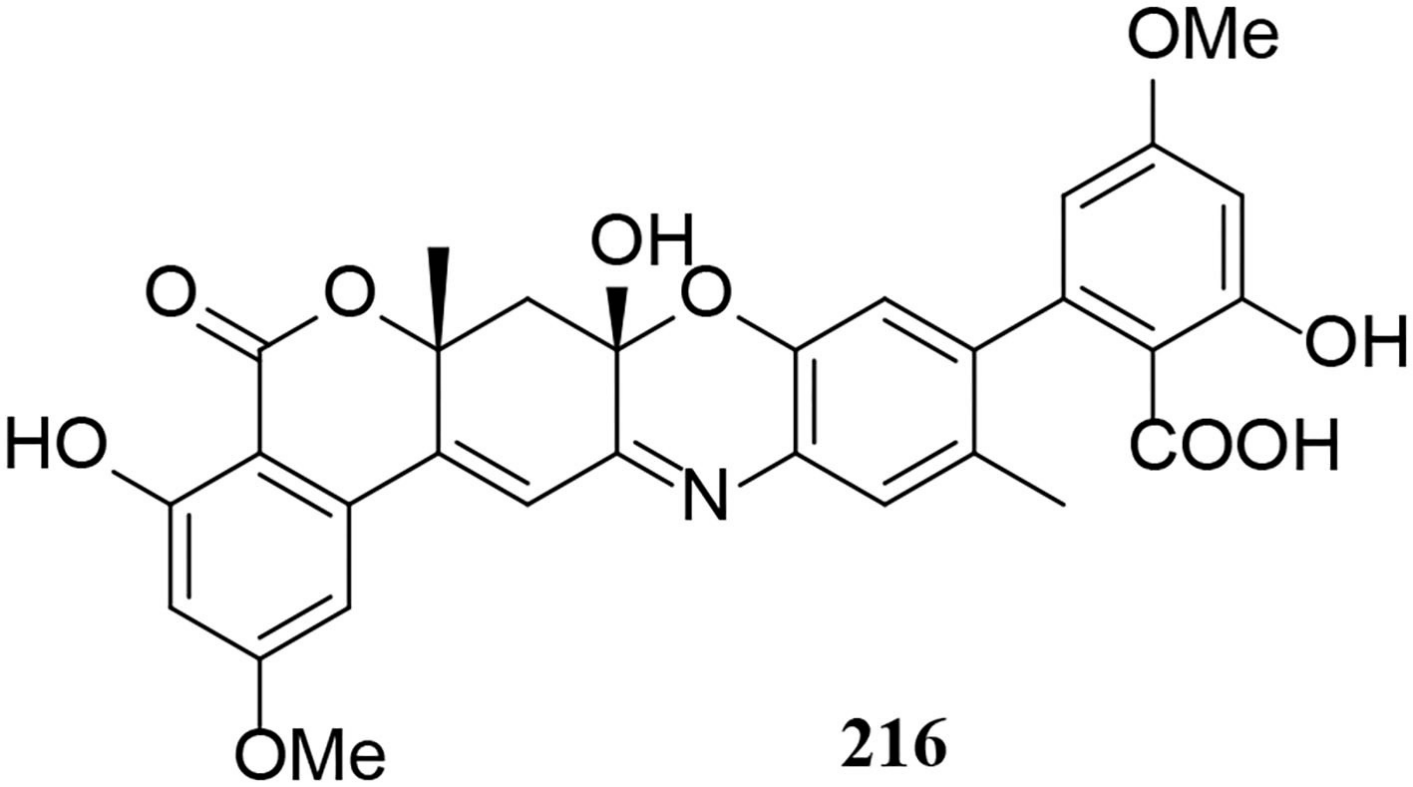
Other derivative (**216**) from *Alternaria* fungi.

One miscellaneous metabolite **216** was isolated from *Alternaria* fungi (Figure 13). Notably, bialternacins A (**216**) is a racemic mixture of aromatic polyketone dimer with an unprecedented 6/6/6/6/6/6-hexacyclic scaffold (Yang C. L. et al., 2019).

## 3. Biological activity

The biological activities of secondary metabolites of *Alternaria* fungi are listed in Table 1. As shown in Table 1, antitumor, antibacterial, and antioxidant properties were characterized as the main indexes to assess the biological activity of these natural products (Zhang et al., 2021). Detailed descriptions of the compounds with excellent biological activities are provided as follows.

**Table 1:** Bioactivities and sources of secondary metabolites from *Alternaria* fungi.

**Compounds**	***Alternaria* species**	**Source of strain**	**Biological activities**	**References**
**Polyketides**				
Alternaritins B–C (**1**–**2**)	*Alternaria* sp. MG1	*Vitis quinquangularis*	Moderate inhibition of COX-2	Tian et al., 2021
Alternaria A (**4**), Alternaria C (**6**), Alternaria F (**9**)	*Alternaria* sp. HJT-Y7	*Rhodiola tibetica*	Anti-SARS-CoV- 2 virus	Lu et al., 2021
(4S,5S)-Alterpyrone A (**10**a), (4R,5R)-Alterpyrone A (**10**b)	*A*. *brassicicola*	*Siegesbeckia pubescens* Makino	Herbicidal activity	Li et al., 2021
Alternariol-9-methyl ether (**19**)	*Alternaria* sp. LV52	*Cystoseira tamariscifolia*	Significant cytotoxicity	Mahmoud et al., 2021
Alternate (**22**)	*A*. *alternata*	*Paeonia lactiflora*	Moderate cytotoxicity	Wang et al., 2021
Alternatain D (**27**)	*A*. *alternata* MT-47	*Huperzia serrata*	Inhibition of platelet ATP release	Yang H. et al., 2019
(+)- and (–)-Alternamgin (**29**)	*Alternaria* sp. MG1	*Vitis quinquangularis*	Moderate cytotoxicity	Wu J. C. et al., 2019
Bialternacin E (**30**)	*Alternaria* sp. NF2128	*Maianthemum bifolium*	Inhibition of acetylcholinesterase	Yang C. L. et al., 2019
(+)-(S)-6-hydroxy-1,8-dimethoxy-3a-methyl-3,3a-dihydrocyclopenta[c]-isochromene-2,5-dione (**33**a), (–)-(R)-6-hydroxy-1,8-dimethoxy-3a-methyl-3,3a-dihydrocyclopenta[c] isochromene-2,5-dione (**33**b)	*Alternaria* sp. TNXY-P-1	*Arisaema heterophyllum*	Significant selective antitumor	Lu et al., 2018
3-epi-dihydroaltenuene A (**35**)	*Alternaria* sp. Samif01	*Salvia miltiorrhiza* Bunge	Significant antioxidant	Tian et al., 2017
Altenuene-2-acetoxy ester (**37**), Altenuene-3-acetoxy ester (**38**), (+)-(10R)-7-hydroxy-3-(2-hydroxy-propyl)-5, 6-dimethyl-isochromen-1-one (**39**)	*A*. *alternata*	*Camellia sinensis*	Moderate antibacterial	Wang et al., 2014
Isotalaroflavone (**43**)	*A*. *alternata* ZHJG5	*Cercis chinensis*	Significant antibacterial	Zhao et al., 2021
(±)-Alternaone A (**47**)	*A*. *alternata* ZHJG5	*Cercis chinensis*	Moderate antibacterial	Zhao et al., 2020
(±) alternarlactones A (**50**) and B (**51**)	*A*. *alternata* P1210	*Salicornia* sp.	Antiparasitic	Shi et al., 2019
Phomalichenone F (**56**)	*Alternaria* sp. MCCC 3A00467	Deep-sea sediments	Cytotoxicity	Zhong et al., 2022
Alterchromanone A (**59**)	*A*. *longipes*	Mangrove	Antioxidant	Liu et al., 2021
5-chloromoniliphenone (**61**), methyl 3,8-dihydroxy-6-methyl-9-oxo-9H-xanthene-1-carboxylate (**65**)	*A*. *sonchi*	_	Selective inhibition of carboxylesterase	Dalinova et al., 2020
Methyl 3,8-dihydroxy-6-methyl-4-chloro-9-oxo-9H-xanthene-1-carboxylate (**63**), chloromonilinic acid B (**69**)	*A*. *sonchi*	_	Antibacterial, insecticidal	Dalinova et al., 2020
(2'S)-2-(2-acetoxypropyl)-7-hydroxy-5-methylchromone (**73**)	*A*. *brassicae* JS959	*Vitex rotundifolia*	Lipoprotein oxidation inhibitory	Kim et al., 2019
4-chloro-1,5-dihydroxy-3-hydroxymethyl-6- methoxycarbonyl-xanthen-9-one (**74**)	*Alternaria* sp. R6	Mangrove	Antibacterial	Wang J. et al., 2015
Prenylcephalochromin A **(76**), prenylcephalochromin B (**77**), cephalochromin (**78**)	*Alternaria* sp. ZG22	*Dasymaschalon rostratum*	Inhibition of α-Glucosidase	Song et al., 2021
Polluxochrin (**79**), dioschrin (**80**), castochrin (**81**)	*Alternaria* sp.	Soil sample	Antibacterial, weak cytotoxicity	Cai et al., 2014
(±)- (4S*,5S*)-2,4,5-trihydroxy-3-methoxy-4-methoxycarbonyl-5-methyl-2-cyclopenten-1-one (**91**)	*Alternaria* sp.	Mangrove	Significant ABTS scavenging, antibacterial	Wang J. et al., 2015
**Nitrogen-containing metabolites**				
Anthrininones B–C (**98**–**99**)	*A*. *tenuissima* DFFSCS013	Deep-sea sediments	Significant inhibition of IDO1 and of protein tyrosine phosphatase	Pan et al., 2019
3R, 14S-ochratoxin A (**103**)	*A*. *brassicae* 93	*Comanthina schlegeli*	Significant cytotoxicity	Li et al., 2015
(±)- (4R*,5S*,6S*)-3-amino-4,5,6-trihydroxy-2-methoxy-5-methyl-2-cyclohexen-1-one (**106**)	*Alternaria* sp.	Mangrove	Significant ABTS scavenging	Wang J. et al., 2015
Altercrasins D–E (**110**–**111**)	*Alternaria* sp.OUPS-117D-1	*Anthocidaris crassispina*	Significant cytotoxicity	Yamada et al., 2019
Swainsonine (**118**)	*A*. *oxytrop*	Lockfeed	Cytotoxicity	Tan et al., 2019
Indole-3-methylethanoate (**122**)	*A*. *alternate*	*Psidium littorale*	Neuroprotection	Xu et al., 2019
Adenine (**127**), allantoin (**128**)	*Alternaria* sp.	*Nerium indicum*	Antioxidant and antibacterial	Miao et al., 2017
**Quinones**				
Isoxanalteric acid I (**132**)	*Alternaria* sp. MG1	*Vitis quinquangularis*	Moderate COX-2 inhibition and antibacterial	Tian et al., 2021
Altertoxin VII (**133**)	*Alternaria* sp. PfuH1	*Pogostemon cablin*	Antibacterial	Kong et al., 2020
Altertoxin II (**135**)	*Alternaria* sp. LV52	*Cystoseira tamariscifolia*	Cytotoxicity	Mahmoud et al., 2021
Altertoxin I (**136**), altertoxin II (**137**)	*A*. *alternata*	Potato and rice	Cytotoxicity	Hohenbichler et al., 2020
3,6,6a,9,10-pentahydroxy-7,8-epoxy-4-oxo-4,5,6,6a,6b,7,8,9-octahydroperylene (**139**), 3,6,6a,7,10-pentahydroxy-4,9-dioxo-4,5,6,6a,6b,7,8,9-octahydroperylene (**140**)	*Alternaria* sp.	*Pinusponderosa*	Insecticidal, antimalarial, and cytotoxicity	Tantry et al., 2018
Altertoxin VII (**141**)	*Alternaria* sp. SCSIO41014	*Callyspongia* sp. sponge	Cytotoxicity	Pang et al., 2018
3,11α,12β,13β,16-Pentahydroxy-11,12-dihydroperylen-6(13H)-one (**144**)	*Alternaria* sp. NH-F6	Deep-sea sediments	Inhibition of BRD4 protein	Ding et al., 2017
Anthrininone A (**146**)	*A*. *tenuissima* DFFSCS013	Deep sea sediments	Effect of calcium ion level and IDO1	Pan et al., 2019
Macrosporin (**147**)	*Alternaria* sp. WZL003	Gorgonian *Echinogorgia rebekka*	Significant antibacterial	Wang Y. N. et al., 2015
Alterporriol T (**150**)	*Alternaria* sp. XZSBG-1	Carbonate saline lake	Cytotoxicity	Chen et al., 2014
(+)-aS-alterporriol C (**153**)	*Alternaria sp*. SK11	Mangrove	Anti-mycobacterium tuberculosis	Xia et al., 2014
Altersolanol **(154**)	*Alternaria* sp.	*Erythrina variegata*	Antiangiogenic	Pompeng et al., 2013
**Terpenoids**				
Oxytropiol A (**156**)	*A*. *oxytropis*	*Oxytropis glabra*	Cytotoxicity	Tan et al., 2019
Sesteralterin (**170**)	*A*. *alternata* k21-1	*Lomentaria hakodatensis*	Phytotoxicity	Shi et al., 2017
Alterbrassicicene B (**172**), 3-Ketobrassicicene W (**173**), 1β,2β-Epoxybrassicicene I (**175**), Alterbrassicicene E (**177**)	*A*. *brassicicola*	*Siegesbeckia pubescens Makino*	Weak cytotoxicity, moderate anti-inflammatory effect	Li et al., 2020a
Alterbrassinoids A–D (**178**–**181**)	*A*. *brassicicola*	_	Cytotoxicity	Li et al., 2019a
Alterbrassicenes B–D (**182**–**184**)	*A*. *brassicicola*	*Siegesbeckia pubescens Makino*	Moderate cytotoxicity	Li et al., 2020b
Alterbrassicicene A (**185**)	*A*. *brassicicola*	*Siegesbeckia pubescens Makino*	PPAR- γ agonist	Li et al., 2018
Alterbrassicene A (**186**)	*A*. *brassicicola*	_	IKK β inhibitory	Hu et al., 2018
Tricycloalternarenes Q–W (**187**–**193**)	*A*. *brassicicola*	*Siegesbeckia pubescens* Makino	Selective cytotoxicity	Li et al., 2019b
17-O-methyltricycloalternarene D (**194**), methyl nortricycloalternarate (**195**)	*Alternaria* sp. k21-1	A marine red alga-epiphyte	Inhibition of marine plankton growth	Shi et al., 2018a
2H-(2E)-tricycloalternarene 12a (**196**)	*Alternaria* sp. W-1	*Laminaria japonica*	Cytotoxicity	Shen et al., 2018
Tricycloalterfurenes A–D (**197**–**200**)	*A*. *alternata* k21-1	*Lomentaria hakodatensis*	Inhibition of marine plankton growth	Shi et al., 2017
15-hydroxytricycloalternarenes (**201–202**)	*A*. *alternata* k23-3	Marine alga	Inhibition of marine plankton growth	Shi et al., 2018a
Alternarin A (**203**)	*Alternaria sp*. ZH-15	Lobophytum crassum	Neuroprotective	Wang H. L. et al., 2020
Tricycloalternarene X (**204**)	*Alternaria* sp. JJY-32	*Callyspongia* sp.	Cytotoxicity	Wang L. et al., 2020
Tricycloalternarene 3b (**210**)	*A. tenuissma* DFFSCS013	The deep sea	Antibacterial	Pan et al., 2018
Tricycloalternarene 3a (**214**), Tricycloalternarene F (**215**)	*Alternaria* sp. Be-1	*Pierisrapae Linne*	Significant tyrosine kinase inhibitory	Zhang et al., 2015

### 3.1. Antibacterial activity

Pyranones (**37–39**, **43**, **47**) can effectively inhibit fungal growth and have a great impact in the application of biofungicide. Of these, (+)-**37** and (+)-**38** showed a productive inhibitory effect on *Candida albicans* with IC_50_ of 19.5 ± 1.5 and 24.0 ± 1.0 μg/ml, while (–)-**37** and (–)-**38** were less active, suggesting different antifungal abilities between enantiomers (Wang et al., 2014). Notably, pyranone (**43**) showed significant activities toward the phytopathogenic bacteria *Xoo, Xanthomonas oryzae pv. oryzicola* (*Xoc*), and *Rs* with minimal inhibitory concentration (MIC) value of 0.5–64 μg/ml, indicating the potential of **43** for the development of novel bactericides (Zhao et al., 2021). Similarly, enantiomeric dibenzo-α-pyrone derivative **(47**) exhibited moderate antibacterial activities on phytopathogenic bacteria *Xoo* and *Xoc* with MIC value of 32–100 μg/ml (Zhao et al., 2020). Pyranone (**63**) exhibited antimicrobial activity toward *Bacillus subtilis* and *Candida tropicalis* with MIC of 0.5–5 μg/disk, which proved that they may be effective biological probes for antibacterial agents (Dalinova et al., 2020). γ-pyranones **79–81** inhibited methicillin-resistant *Staphylococcus aureus* (MRSA) with an MIC of 2.9, 3.2, and 2.0 μg/ml, respectively (Cai et al., 2014). The structure–activity relationship (SAR) of **79–81** indicated that the possible intramolecular cyclization caused by sulfur atom was necessary. Pyranones **74** and **91** showed antibacterial activity against *Fusarium graminearum* with MIC values of 107.14 and 215.52 μM, respectively (Wang J. et al., 2015). Compared with **91**, **74** showed better activity, probably due to the presence of chlorine atoms in molecular. Moreover, perylenequinone (**133**) showed antibacterial activity against *Streptococcus agalactiae*, with an MIC of 17.3 μg/ml (Kong et al., 2020). Moreover, anthraquinone (**147**) had a strong inhibitory effect on *Vibrio anguillarum* with an MIC value of 17.6 μmol/L, which can destroy the cell wall and cell membrane, and its effect was equivalent to that of streptomycin at the same concentration (Wang Y. N. et al., 2015). In antimicrobial and antifungal activity tests, meroterpenoid (**210**) showed a significant inhibitory effect on *E. coli* and *B. subtilis* (Pan et al., 2018). Ethyl acetate (EA) fraction of endophytic *A. tenuissima* OE7 had an inhibitory effect on *C. albicans* (Chatterjee et al., 2020). Two fractions that could inhibit α-glucosidase activity were obtained from *Alternaria destruens*, which showed broad-spectrum antibacterial activity (Kaur et al., 2020). The *Alternaria* extracts with excellent antibacterial activity provide an important direction for future research on antibacterial drugs and will guide bioactivity isolation.

### 3.2. Antioxidant activity

Antioxidants acknowledged as “free-radical scavengers” have been widely connected to the treatment of aging, cancer, diabetes, etc., (Neha et al., 2019). Pyranone (**59**) showed scavenging activity of 1,1-diphenyl-2-picrylhydrazyl (DPPH) radical, with an IC_50_ of 56.3 μg/ml (Liu et al., 2021). Compounds **91** and **106** showed strong free-radical scavenging efficiency for 2,2′-azino-bis (3-ethylbenzthiazoline-6-sulphonic acid) (ABTS) with EC_50_ values of 8.19 ± 0.15 and 16.09 ± 0.01 μM, respectively, which were stronger than that of the positive control ascorbic acid (EC_50_, 17.14 ± 0.11 μM) (Wang J. et al., 2015). A free-radical scavenging test showed that pyranone (**35**) and nitrogenous metabolites (**127**–**128**) also had significant antioxidant activity (Miao et al., 2017; Tian et al., 2017). The discovery of antioxidant compounds is of great significance to various nutraceuticals and cosmetic medicine industries, which has been widely considered as a promising source of new therapeutics.

### 3.3. Enzyme-inhibitory metabolites

Inhibitory enzymes are often used as biocatalysts to participate in the catalysis of various metabolism activities in living organisms. They attach to the enzyme's active site and reduce the its activity, which can be used as medicine, pathogens, or insecticides in biotechnological applications. Pyranones (**1**–**2**) and perylene quinone (**132**) showed a moderate inhibitory effect on cyclooxygenase-2 (COX-2), with IC_50_ of 1.50, 7.00, and 7.00 μM. For comparison, celecoxib showed IC_50_ values of 0.06 μM as a positive control, demonstrating their potential for pharmaceutical uses in antipyretic analgesic and anti-inflammatory drugs (Tian et al., 2021). Pyranone (**27**) showed antiplatelet and anticoagulant effects after intracoronary tent implantation, with an IC_50_ of 57.6 ± 3.2 μM (Yang H. et al., 2019). Pyranone (**30**) showed an inhibitory effect on acetylcholinesterase with an IC_50_ of 15.5 μM (Yang C. L. et al., 2019). Huperzine can also inhibit acetylcholinesterase activity, which can be a prospective therapeutic drug candidate for Alzheimer's disease (Zaki et al., 2019). In addition, compounds **61** and **65** displayed selective carboxylesterase inhibition activity at a concentration of 100 μg/ml as a key serine hydrolase with potential applications in the treatment of hypertriglyceridemia, obesity, and type 2 diabetes (Zou et al., 2018; Dalinova et al., 2020). Pyranones (**76–78**) and anthraquinone (**150**) showed inhibitory activity on α-glucosidase activity with IC_50_ values of 2.9, 2.8, 3.1, and 7.2 μM, respectively, indicating that they have potential in the treatment of diabetes (Chen et al., 2014; Ruiz-Vargas et al., 2019; Song et al., 2021). Notably, anthrinones A-C (**146**, **98–99**) showed significant inhibitory activity on indoleamine 2,3-dioxygenase 1 (IDO1), and amides (**98**–**99**) had selective inhibitory activity on different protein tyrosine phosphatases (Pan et al., 2019). Comparatively, anthraquinone (**153**) showed strong inhibitory activity against *Mycobacterium tuberculosis* protein tyrosine phosphatase B (MptpB), with IC_50_ of 8.70 μM (Xia et al., 2014). Similarly, meroterpenoids (**214**–**215**) showed strong inhibitory activity on three tyrosine kinase (EGFR, VEGFR-1, and c-Met) with an inhibition rate of 28.4–56.2%, indicating stronger activity than that of the positive control erlotinib, pazopanib, and bms-777607 (inhibition rate, 100.2, 98.5, and 99.1%, respectively) (Zhang et al., 2015). However, alternative monomer ether (AME) showed selective inhibitory activity on monoamine oxidase A (MAO-α), which may be related to dibenzo of α-pyranone (Lee et al., 2017). The cytotoxin produced by *Alternaria* can also inhibit topoisomerase (Jarolim et al., 2017).

### 3.4. Antitumor activity

Some *Alternaria* metabolites that have been identified as cytotoxic are considered potential sources of cancer chemo-preventive agents. Pyranone **19** and perylene quinone **135** on A549 (EC_50_, 0.73, 0.40 μg/ml) and PC3 (EC_50_, 0.17, 0.12 μg/ml) cells exhibited potential cytotoxicity *in vitro* (Mahmoud et al., 2021). Pyranones **22** and **29** exhibited moderate cytotoxicity against different tumor cells (MDA-MB-231, MCF-7, HeLa, and HepG2), where compound **22** was the most active in MDA-MB-231 and MCF-7 with IC_50_s of 20.1 and 32.2 μM, respectively (Wu J. C. et al., 2019; Wang et al., 2022). Notably, one pair of new cyclopentaisochromenone enantiomers, (+)-**33**a and (–)-**33**b from *Alternaria* sp. TNXY-P-1, showed distinct selective antitumor activities against HL-60 cell lines with IC_50_ values of >200 and 75.3 μM, respectively (Lu et al., 2018). Pyranone (**56**) exhibited cytotoxicity to human myeloma cancer U266, with an IC_50_ of 24.99 μg/ml (Zhong et al., 2022). However, γ-pyranones **79**–**81** exhibited weak cytotoxicity to pancreatic cancer cells (MIA PaCa-2), with IC_50_s of 50.8, 30.3, and 29.3 μM, respectively (Cai et al., 2014). Amide **103** has certain cytotoxicity, strong nephrotoxicity, neurotoxicity, immunotoxicity, carcinogenicity, teratogenicity, and mutagenicity (Li et al., 2015). In comparison, the cytotoxicity of amides (**110**–**111**) was equivalent to that of 5-fluorouracil (Yamada et al., 2019). Alkaloid (**118**) was merely cytotoxic to A549 and HeLa, with IC_50_s of 10.93 ± 0.80 and 66.69 ± 1.58 μM, respectively (Tan et al., 2019). Antitumor activity of **118** to A549 is equivalent to the positive control cis-platinum (IC_50_ values of 8.73 ± 1.77) (Tan et al., 2019). Two variants of an extract from cultured *Alternaria alternata*, quinones **136**–**137**, displayed dose-dependent enhancements of cytochrome P450 (CYP) activity by testing singularly the 7-ethoxy-resorufin-O-deethylase (EROD) assay in MCF-7 breast cancer cells (Hohenbichler et al., 2020). In addition, perylenequinone (**141**) had cytotoxicity to K562, SGC-7901, and BEL-7402 with IC_50_s are 26.58 ± 0.80, 8.75 ± 0.13, and 13.11 ± 0.95 μg/ml, respectively (Pang et al., 2018). Diterpenes **172**, **173**, **175**, and **177** were active against certain human tumor cell lines, with IC_50_ values ranging from 25.0 to 38.2 μM, but had no obvious toxicity to the normal LO2 cells (Li et al., 2020a). Interestingly, terpenoids **178**–**184**, **187**, **188**, **191**, and **193** all had antitumor activity, of which diterpenes **178**–**181** exhibited moderate cytotoxicity to OCvar, MDA-MB-231, HeLa, and HT-29, while being non-toxic to normal cells (Li et al., 2019a). Diterpenes **182**–**184** exhibited moderate cytotoxic activity against certain human tumor cell lines, with IC_50_ values in the range of 15.87–36.85 μM, but no obvious cytotoxicity to human normal cell LO2 (Li et al., 2020b). Meroterpenoids **187**, **188**, **191**, and **193** exhibited selective cytotoxicity to some human cancer cells, with IC_50_s ranging from 12.83 to 32.87 μM; meanwhile, they had no obvious effect on normal human LO2 cells, indicating their significant potential as selective cancer chemo-preventive agents (Li et al., 2019b). Meroterpenoid **196** displayed inhibitory activity against the growth of SMMC-7721 cells with an IC_50_ of 49.7 ± 1.1, which is comparable with that of the positive control, cisplatin (IC_50_ = 6.5 ± 0.5 μg/ml) *(*Shen et al., 2018). Similarly, meroterpenoid **204** showed cytotoxicity to HL-60 and HO8910 cells, with IC_50_ of 7.54 and 20.32 μM (Wang L. et al., 2020). The emergence of a large number of metabolites with antitumor activities provides more opportunities for the development of cancer-treatment drugs.

### 3.5. Phytotoxicity

Partial metabolites of *Alternaria* fungi have exhibited pathogenicity that causes damage to plants and possess the potential to be as herbicides on account of excellent phytotoxicity (Meena and Samal, 2019; Leyte-Lugo et al., 2020). In phytotoxicity assays, pyranone **10**a and **10**b showed a significant inhibition rate on the germination of monocotyledonous weed seeds (*E. crusgalli* and *S. viridis*), with inhibitory ratios ranging from 68.6 ± 6.4 to 84.2 ± 5.1%, which was equivalent to that of the positive control, glyphosate, at a concentration of 100 μg/ml (Li et al., 2021). At 1 mg/ml, pyranone **69** showed contact insecticidal activity against wheat aphids (*Schizaphis graminum*), indicating its use as a potential agricultural insecticide (Dalinova et al., 2020). In addition, sesquiterpenoid **156** showed an inhibition of the root growth of *Arabidopsis thaliana* but no remarkable effect on leaf growth (Tan et al., 2019). Sesquiterpene (**170**) and meroterpenoids **194**–**195** and **197**–**202** showed weak or moderate inhibition of the growth of marine algae and plankton (Shi et al., 2017, 2018a). Among the three tested marine phytoplankton (*Chattonella marina, Heterosigma akashiwo*, and *Prorocentrum donghaiense*), compounds **170** and **197**–**200** appeared more sensitive to *C. marina* (Shi et al., 2017). Compounds **170** and **197** showed inhibition of these three phytoplanktons but were inactive to the zooplankton *A. salina*, indicating that the hydroxy group positions on ring C had almost no effect on their activities. Hydroxylation at C-2 and C-3 (**199** and **200**) slightly reduced the inhibition of the three phytoplankton (17–56% inhibition) (Shi et al., 2017). Taking structure into account, α-pyranones and terpenoids have great potential as biological control candidates in the application of herbicide, insecticide and marine protection.

### 3.6. Other activities

The various activity of *Alternaria* metabolites is of great significance for research. Pyranones **4**, **6**, and **9** exhibited inhibitory activities related to the SARS-CoV-2 virus (EC_50_ = 0.02, 0.3, 0.07 μM), which is conducive for the development of antiviral drugs (Lu et al., 2021). In addition, pyranones **50**–**51** exhibited a specific inhibitory effect on *L. donovani* and *P. falciparum* (Shi et al., 2019). Interestingly, compounds **139** and **140** have insect-resistant activity, and **139** showed antibacterial activity against *Leishmania donovani* with IC_50_ = 2.55 μg/ml (Tantry et al., 2018). In the study of biological mechanisms, **73** inhibited the oxidation of human plasma high-density lipoprotein (HDL) and low-density lipoprotein (LDL) induced by Cu^2+^, which is of great significance for Cardiovascular and cerebrovascular drugs development (Kim et al., 2019). Compound **144** exhibited a potent inhibition rate of 88.1% at a concentration of 10 μM, which provides new bromodomain protein 4 (BRD4) inhibitors possessing potential antitumoral, antiviral and anti-inflammatory pharmaceutical effects (Ding et al., 2017). In addition, anthraquinone (**154**) was further characterized to have good anti-angiogenic activity *in vivo* and *in vitro* by aortic-sprouting assay in rats, related to inhibited proliferation, tube formation, and migration in endothelial cells (Pompeng et al., 2013). Compounds **122** and **177** exhibited neuroprotective effects and moderate anti-inflammatory effects, respectively (Shi et al., 2017; Tian et al., 2021). Diterpene (**185**) was the first fusicoccane-derived diterpenoid to function as a potent peroxisome proliferator-activated receptor (PPAR-γ) agonist (EC_50_ = 744.1 nM) (Li et al., 2018). In addition, diterpenes (**186**) can inhibit IKKβ in the NF-κB signal pathway and have obvious anti-inflammatory activity (Hu et al., 2018). Meroterpenoid **203** can inhibit neuronal excitation due to its unique cyclopentanone structure, which will be applied in antiepileptic drugs development (Wang H. L. et al., 2020).

## 4. Possible biosynthesis mechanism of secondary metabolites

Biosynthesis is indispensable in the application of natural products. The diversity of endophytic biosynthesis often depends on the diversity of the host and the complexity of its metabolism, which provide a new way for the biosynthesis of various novel compounds (Lin et al., 2019; He et al., 2021). The study of biosynthetic pathways in pharmaceutical chemistry contributes to the discovery of novel drugs and provides new research opportunities for the sustainable development and utilization of natural drugs (Lin et al., 2019; He et al., 2021).

**Figure 14:**
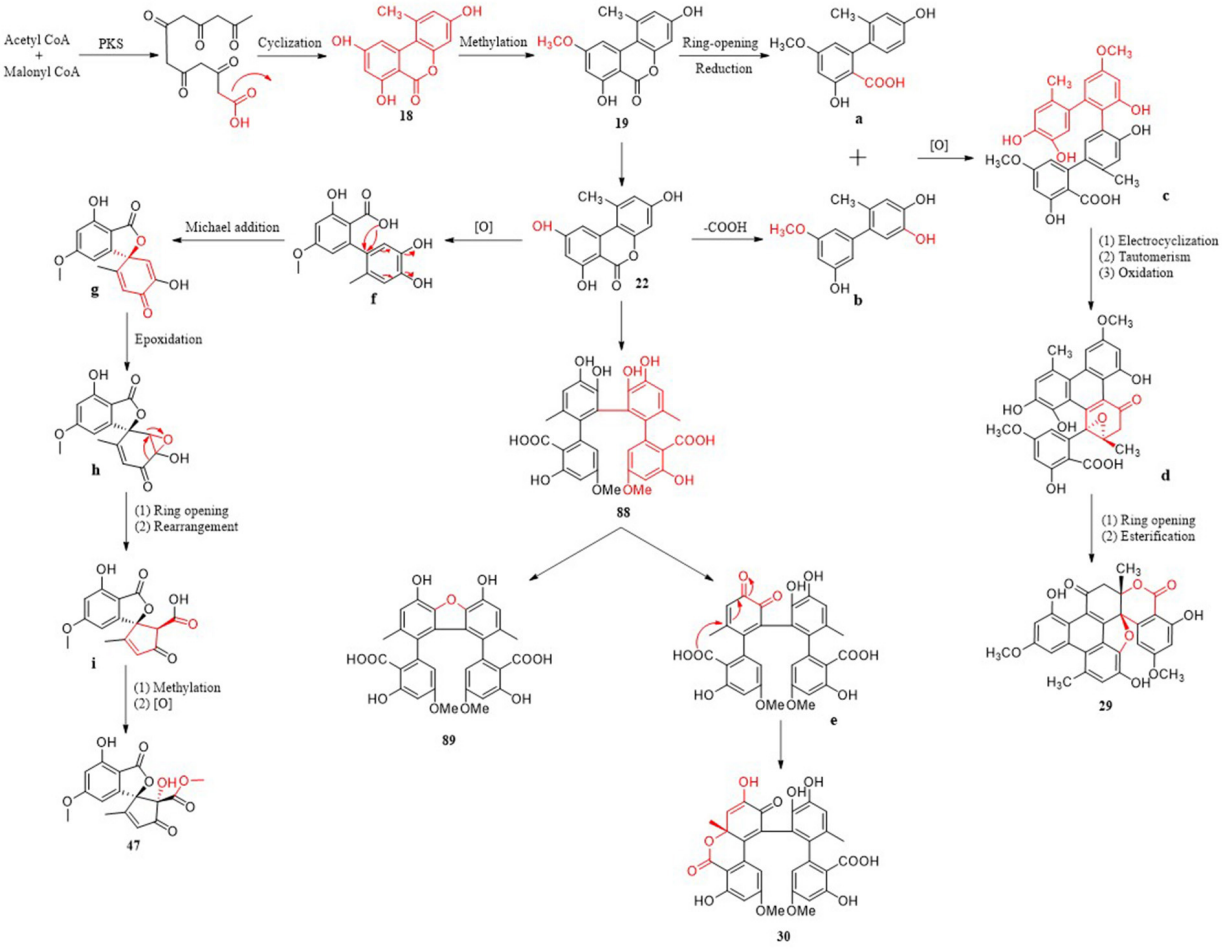
Possible biosynthetic pathway of polyketones.

Polyketones have a variety of structural types and corresponding biosynthetic pathways. Three metabolic pathways of polyketones from *Alternaria* fungi are briefly described, and eight important metabolites are involved (Figure 14). The cinnamic acid–shikimic pathway, a familiar biosynthetic pathway, emerged as the basis of various biosynthetic pathways. Firstly, a heptapeptide intermediate can be produced by iterative condensation of acetyl-CoA(starter) with six molecules of malonyl-CoA (extenders) by polyketide synthase (PKS). Subsequently, the heptapeptide intermediate is cyclized to obtain compound **18**, followed by methylation to obtain **19**. The key intermediate molecule **22** is obtained from the loop-opened of **19**, and then the carboxyl group is removed to form intermediate molecule **b** (Wu J. C. et al., 2019). Finally, compound **29** featuring an unprecedented seven-ring backbone, which was obtained from two molecular intermediates **a** and **b** through oxidative coupling, electrocyclization, tautomerism, oxidation, ring opening, and esterification (Wu J. C. et al., 2019). Complex compounds **30**, **91**, and **92** are also polymerized from two molecules with simple structures. Compound **22** can be dimerized *via* a C–C bond to form compound **88** through intermolecular oxidative phenol coupling, catalyzed most likely by a P450 monooxygenase or laccase. Dehydration of **88** gives compound **89** (Yang C. L. et al., 2019). Oxidation, regioselective intramolecular Michael additions, and Ketone–enol tautomerization of catechol in **88** afforded a new compound, **30**, with a lactone ring (Yang C. L. et al., 2019). It is worth noting that a third possible biosynthetic pathway generates two five-membered rings, which are completely different from the first two pathways. **f** as an ortho-quinone intermediate is formed *via* oxidization of the catechol moiety in **22**, followed by regioselective Michael additions that give intermediate **g**. Intermediate **i** was obtained after epoxidation and stereospecific acid-catalyzed rearrangement of intermediate **g**, indicating that the carbon skeleton of **47** was formed by the key epoxy-rearrangement step (Zhao et al., 2020). Then, compound **47** yielded the methylation and oxidization of **i**. As the starting materials of various metabolic pathways, compound **22** plays an important role in the biosynthesis and transformation of new compounds. This provides a new synthetic route for obtaining the novel structure of *Alternaria* fungi metabolites. In addition, the polyketide metabolites may also have a variety of metabolic pathways to be discovered, which is worthy of deep research.

**Figure 15:**
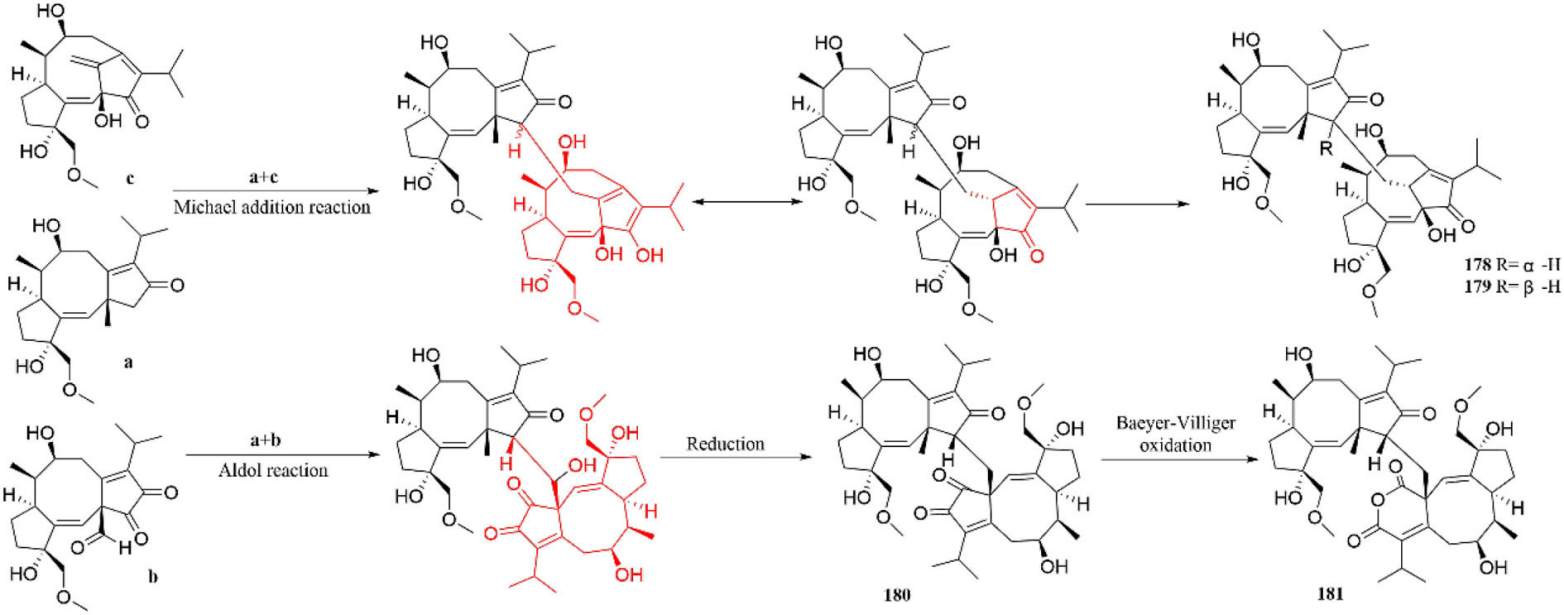
Possible biosynthetic pathway of terpenoid dimer.

Furthermore, the possible biosynthetic pathways of terpenoid dimers are also described (Figure 15). Brassicicene A synthesizes three intermediates (a\b and **c**) through dehydrogenation, oxidation, and Wagner–Meerwein rearrangement. Intermediates **a** and **c** are formed through Michael addition reaction to produce **178** and **179**. Interestingly, they are a pair of unprecedented heterodimers, bearing dicyclopentane [a, d], cyclooctane, and tricyclo [9.2.1.0] tetradecane diterpenoid subunits (Li et al., 2019a). In addition, compounds **180** and **181** are obtained by a series of aldol and reduction reactions, containing two dicyclopentadiene [a, d] cyclooctane diterpene subunits (Li et al., 2019a).

## 5. Conclusion and prospects

**Figure 16:**
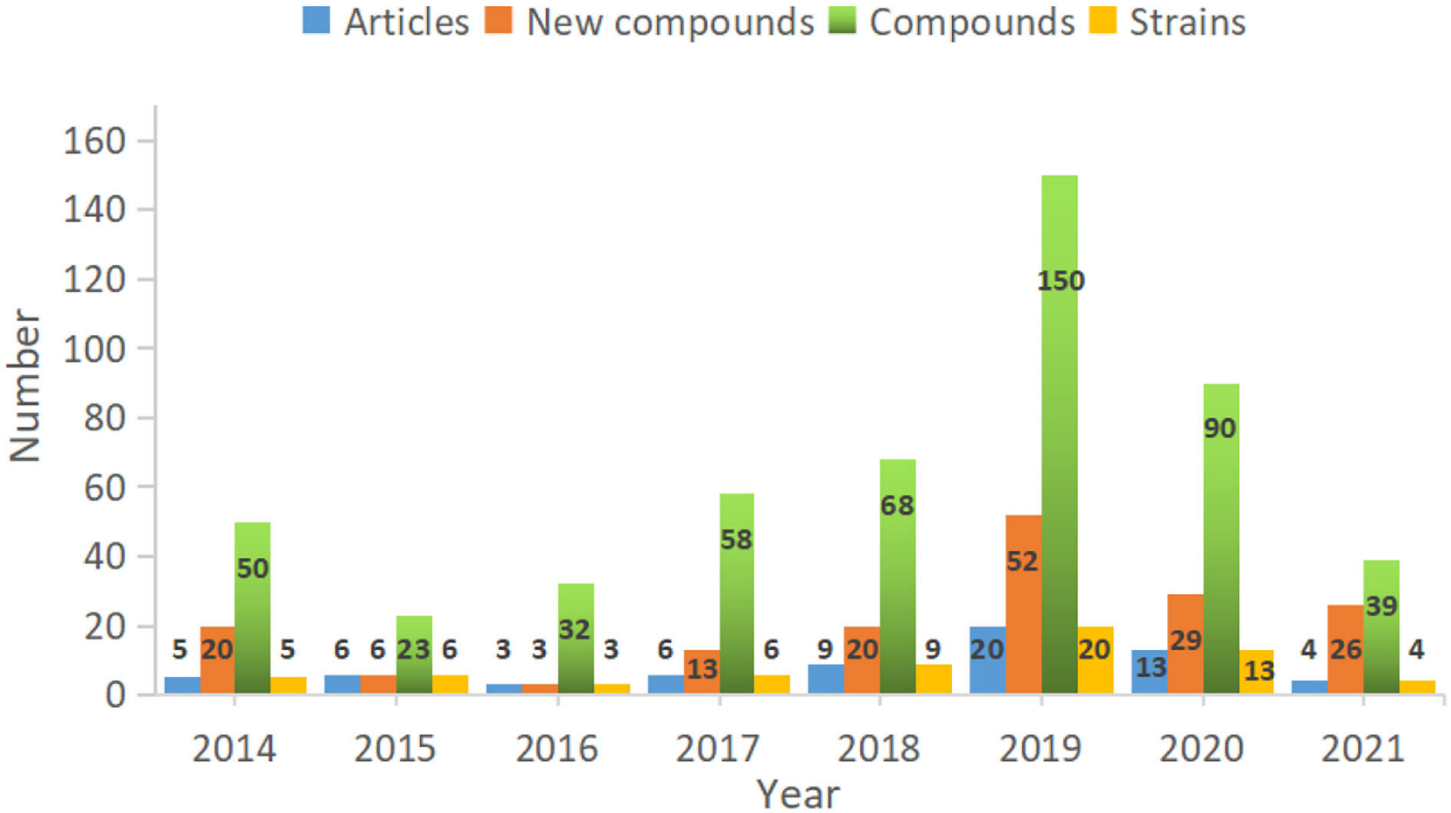
The number of articles, compounds, and strains reported from *Alternaria* fungi in recent years.

**Figure 17:**
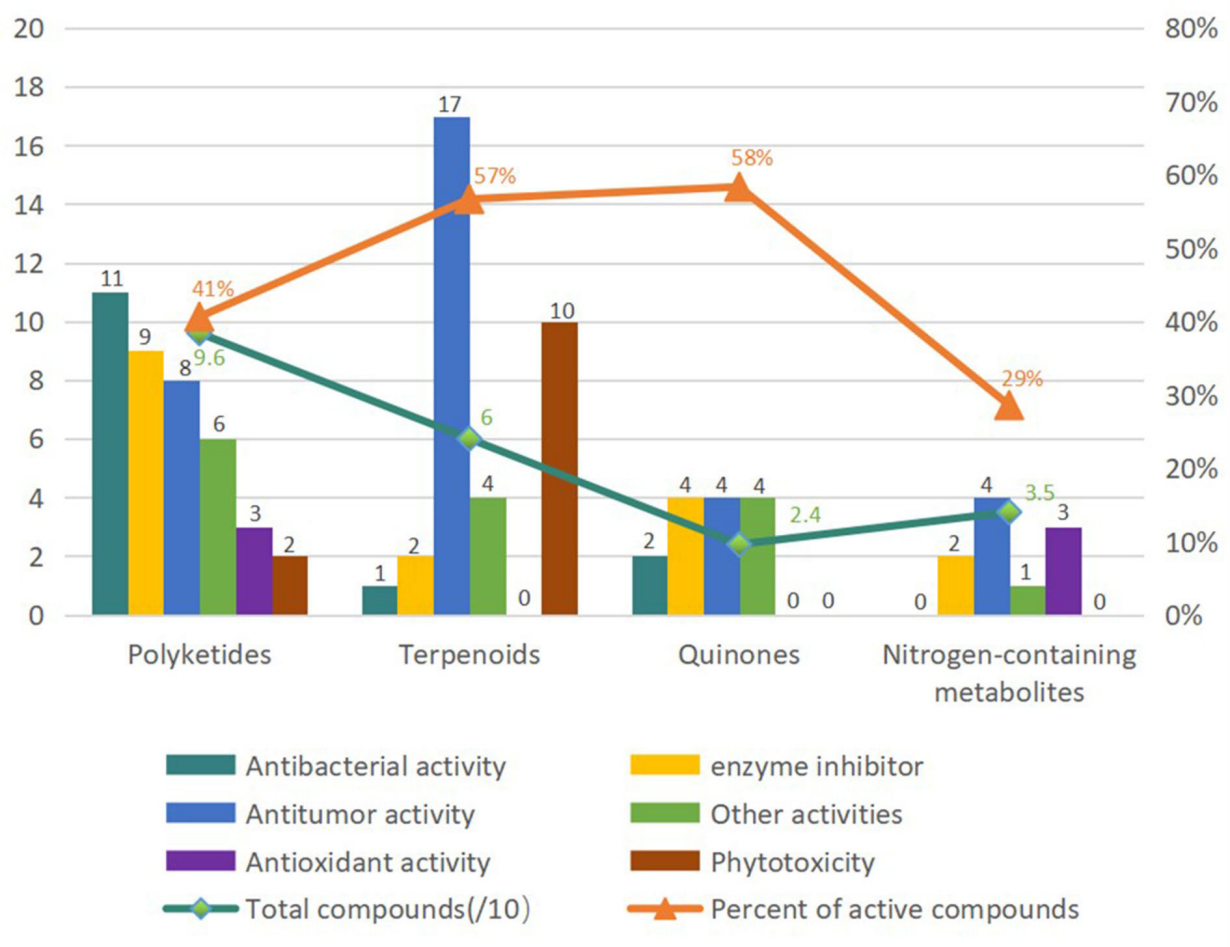
Classification of diverse chemicals of *Alternaria* fungi based on the pharmacological activities.

Fungi are ubiquitous in nature with their tenacious vitality and serve as a wealthy reservoir of structurally diverse metabolites. *Alternaria* fungi occupy a wide spectrum of habitats in diverse ecosystems worldwide. Remarkable progress has been made in the characterization of *Alternaria* fungi metabolites. Data showed that the number of articles published, the number of strains discovered, the number of new compounds, and the total compounds all increased dramatically from 2014 to 2019 (Figure 16). Numerous chemical studies suggest that *Alternaria* fungi are one of the prolific sources of functional biomolecules, including polyketides, terpenoids, quinones, and nitrogen-containing compounds. In this study, 216 metabolites from *Alternaria* species with diverse chemical structures and bioactivities were reviewed based on research from 2014 to 2022 (Figure 17). Polyketones, as the largest number of bio-metabolites, have immense potential in various fields of agriculture and the food and medical industries, considering their characteristics as being antibacterial and enzyme-inhibitory, as well as having antitumor, antioxidant, and phytotoxic properties, amongst others. Remarkably, terpenoids and quinones provided a higher proportion of active compounds. Additionally, the basic biosynthetic pathways of polyketones and terpenoid dimers have also been discussed, which would allow production for industrial purposes.

Unfortunately, the study of secondary metabolites has decreased in the past 2 years. Many metabolites remain to be discovered. Therefore, the construction and breeding of strains, as well as optimization of cultivation and fermentation processes, should be intensively conducted to accelerate the development of valuable products. In addition, a better understanding of the evaluation of bioactivities and pharmacological mechanisms would assist in ascertaining underlying therapeutic potential. Moreover, studying the molecular basis of biosynthetic pathways would be necessary for industrial production. More efforts should be made to explore further sources for the isolation of new *Alternaria* strains and to manufacture novel functional biomolecules using new strategies, such as the “one strain many compounds” (OSMAC) approach, genetic mining (phylogenomic analyses), combined with metabolic engineering.

Finally, we believe the therapeutic potential and chemical diversity of *Alternaria* fungi will provide new avenues for drug discovery with deep research.

## Author contributions

JLi, SY, XY, and JM: conceptualization. SZ, SX, and MR: discussion of the contents. JM, MR, SW, and HZ: writing—original draft preparation. SZ, JLiu, SX, SY, JM, MR, and XY: writing—review and editing. All authors have read and approved the final manuscript.
